# Supplementary material for: Extremely Long C–C Bonds Predicted beyond 2.0 Å
Source: J Phys Chem A. 2023 May 11;127(20):4440–54. doi: 10.1021/acs.jpca.3c01209 (PMC10950299; doi:10.1021/acs.jpca.3c01209)
Supplement: Supplementary file 1 — jp3c01209_si_001.pdf [file jp3c01209_si_001.pdf]

Supporting Information for

EXTREMELY LONG C-C BONDS PREDICTED BEYOND 2.0 Å

Eero J. J. Korpela[1], Jhonatas R. Carvalho[2], Hans Lischka[2], Miklos Kertesz*[1]

[1] Chemistry Department and Institute of Soft Matter, Georgetown University
37th and O Streets, NW, Washington, DC 20057-1227, USA
[2] Department of Chemistry and Biochemistry, Texas Tech University, Lubbock, TX 79409, USA

Table of Contents:

Figure S1: Carbon numbering used for **2A**
Figure S2: Raman spectrum for **2A**
Figure S3: Raman spectrum for **2Atw**
Figure S4: ^{13}C NMR spectra of **1A** and **1Atw**
Figure S5: ^{13}C NMR spectra of **2D** and **2Dtw**
Figure S6: ^{13}C NMR spectra of **10A** and **10Atw**
Figure S7: Rigid scan of ethane comparing ^{13}C NMR chemical shifts and spin densities of C1 and C2 to D_{12}
Table S1: Summary of DFT Calculations
Full list of authors for reference 40 in the main text.

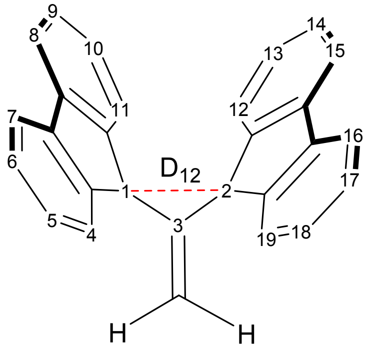

Figure S1. Numbering used to identify carbons on **2A** for NMR and Raman spectra.

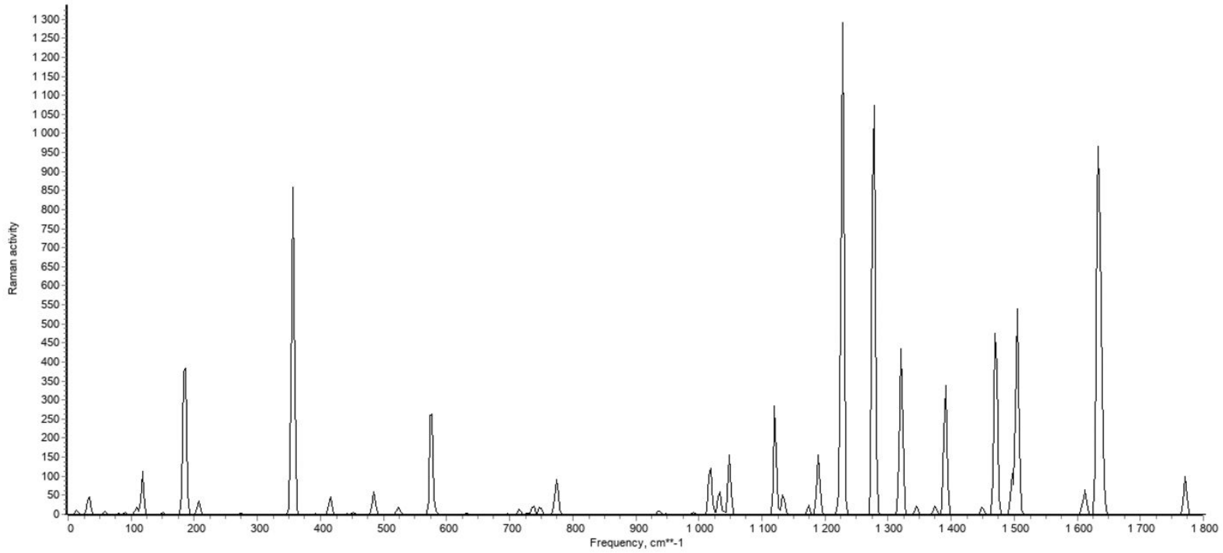

Figure S2. Theoretical Raman vibrational spectra of **2A** (C_{2v}) simulated by the B3LYP-GD3/6-311+G** calculation with the geometry optimized by a B3LYP-GD3/6-311+G** calculation. According to the calculation, the peak of 184 cm^{-1} corresponds to the C1–C2 stretching vibration.

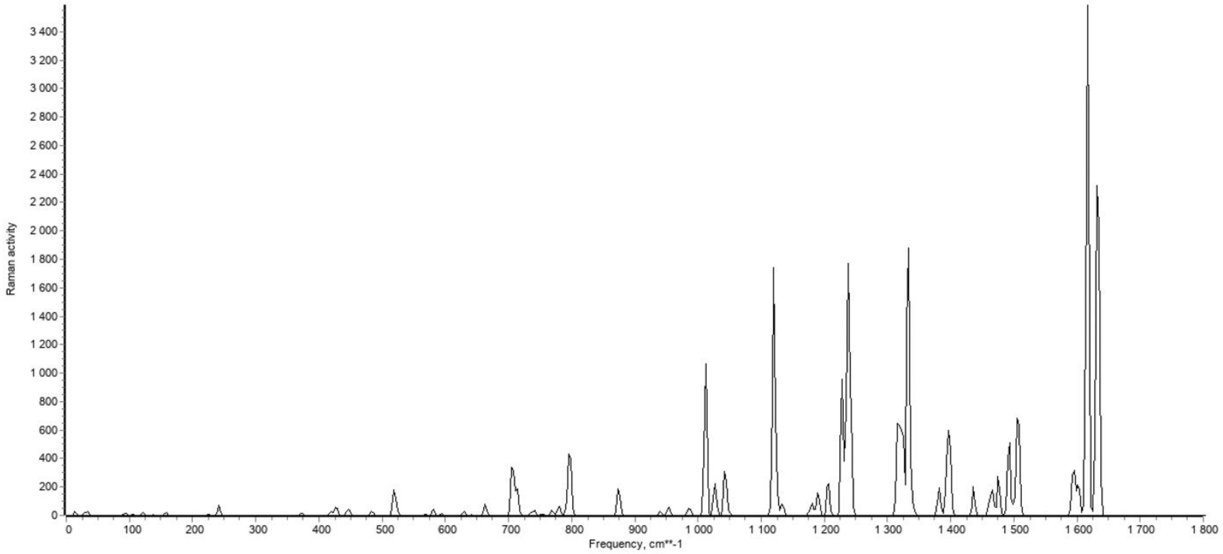

Figure S3. Theoretical Raman vibrational spectra of **2Atw** (C_2) simulated by the B3LYP-GD3/6-311+G** calculation with the geometry optimized by a B3LYP-GD3/6-311+G** calculation.

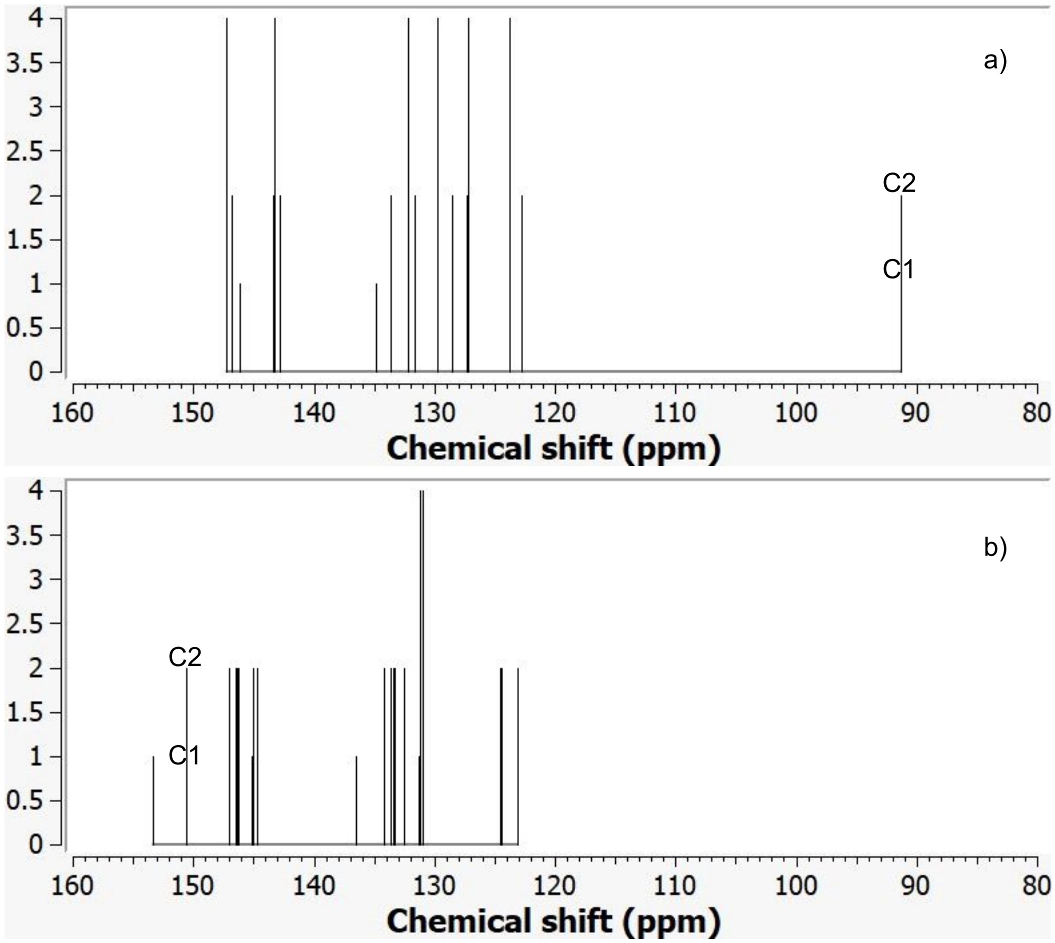

Figure S4. Theoretically predicted ^{13}C chemical shifts of a) **1A** and b) **1Atw** calculated by GIAO-B3LYP-GD3/6-311+G** method. The structures were optimized at the UB3LYP-GD3/6-311+G** level of theory and converged to a C_{2v} symmetry for **1A** and C_2 symmetry for **1Atw**. TSM was computed at B3LYP-GD3/6-311+G** and used as the reference.

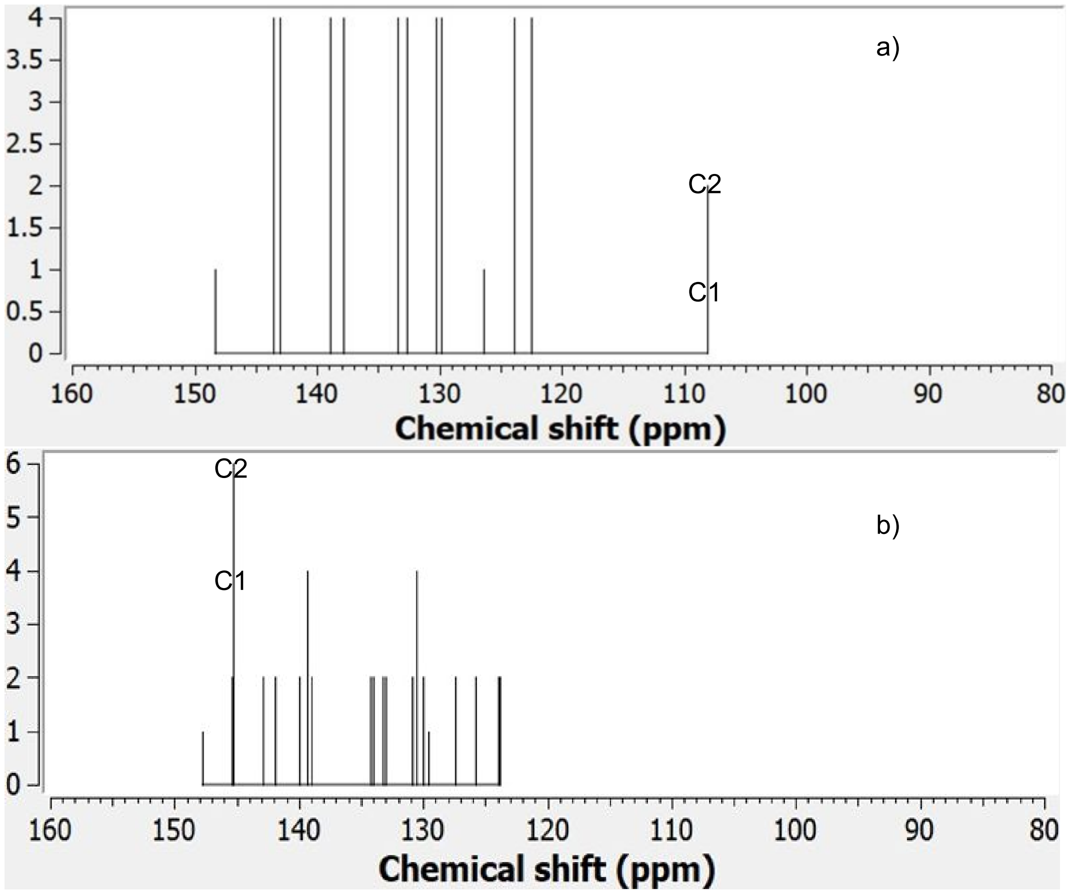

Figure S5. Theoretically predicted ^{13}C chemical shifts of a) **2D** and b) **2Dtw** calculated by GIAO-B3LYP-GD3/6-311+G** method. The structures were optimized at the UB3LYP-GD3/6-311+G** level of theory and converged to a C_{2v} symmetry for **2D** and C_2 symmetry for **2Dtw**. TSM was computed at B3LYP-GD3/6-311+G** and used as the reference.

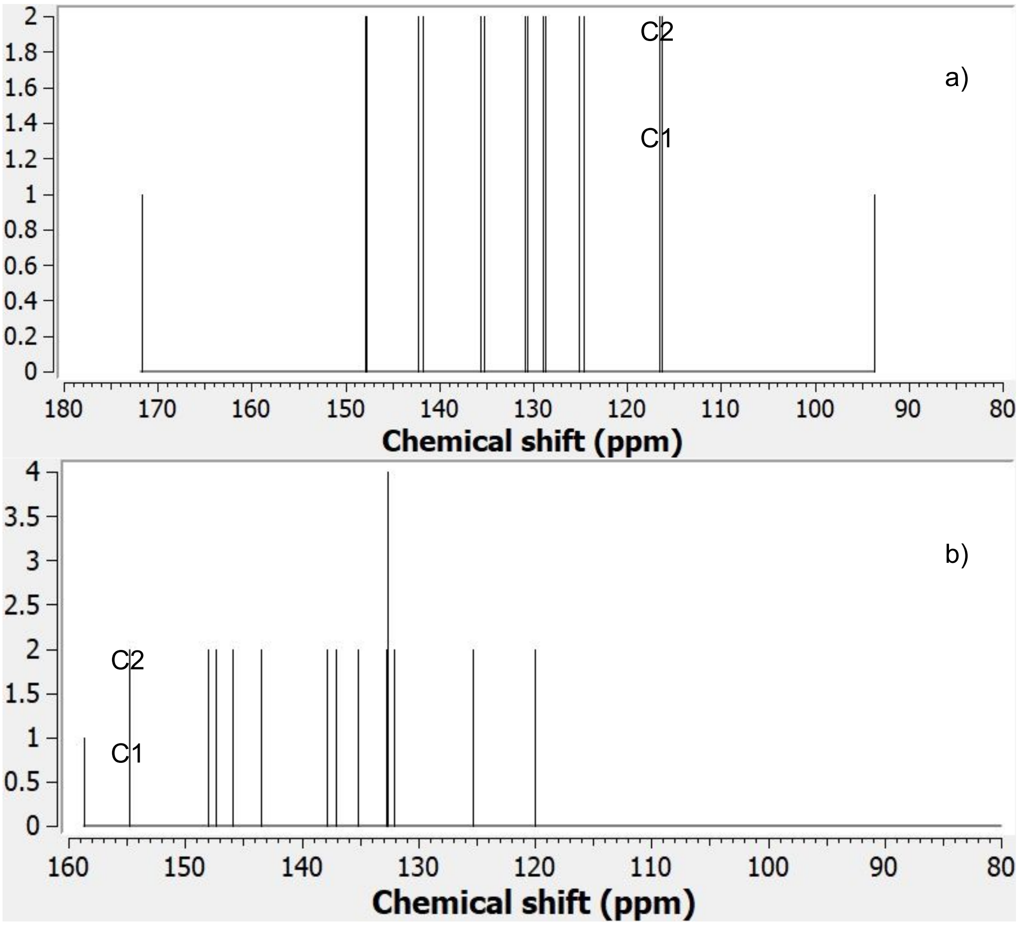

Figure S6. Theoretically predicted ^{13}C chemical shifts of a) **10A** and b) **10Atw** calculated by GIAO-B3LYP-GD3/6-311+G** method. The structures were optimized at the UB3LYP-GD3/6-311+G** level of theory and converged to a C_{2v} symmetry for **10A** and C_2 symmetry for **10Atw**. TSM was computed at B3LYP-GD3/6-311+G** and used as the reference.

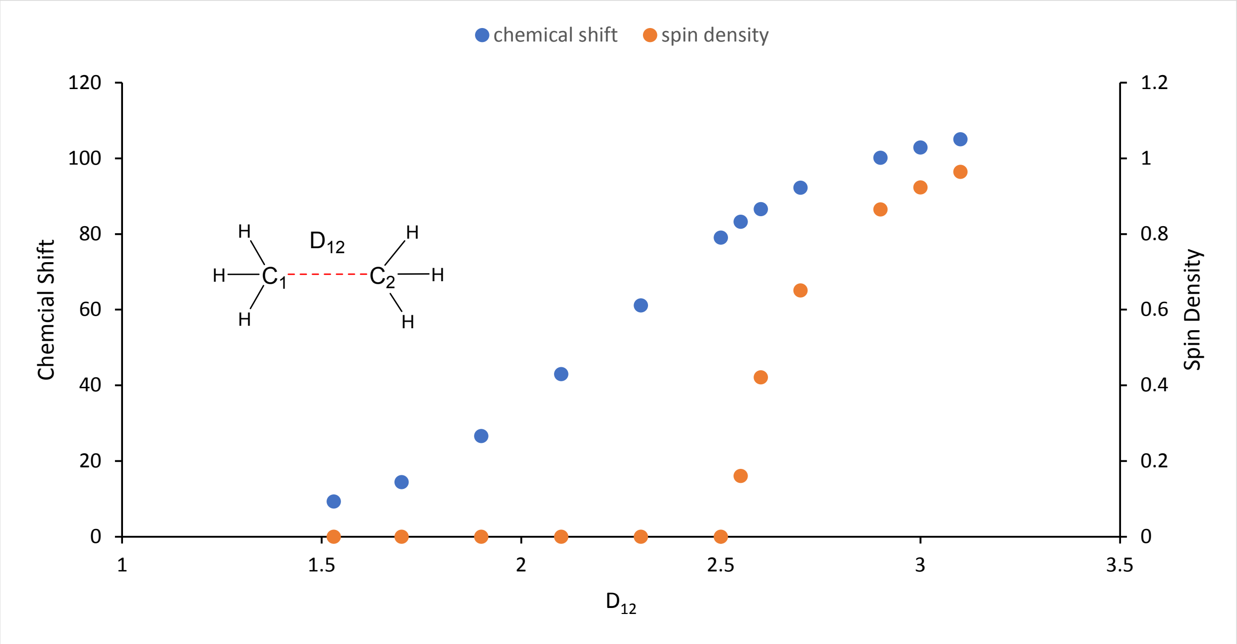

Figure S7. Ethane rigid scan comparing ^{13}C NMR chemical shifts and spin densities of C1 and C2 as a function of D_{12} using GIAO-UB3LYP-GD3/6-311+G**.

Table S1. Cartesian coordinates of target molecules optimized by UB3LYP-GD3/6-311+G** calculations.

1A E(UB3LYP) = -1539.13247903 Hartrees

6	0.507554000	0.000000000	-0.004550000
6	-0.528681000	-1.023919000	-0.001658000
6	-0.528681000	1.023919000	-0.001658000
6	-1.168634000	-1.630035000	-1.169137000
6	-0.840336000	-1.581302000	-2.522029000
6	-1.633586000	-2.278492000	-3.436758000
6	-2.730655000	-3.027037000	-3.005285000
6	-3.036587000	-3.123978000	-1.643115000
6	-2.251078000	-2.438232000	-0.727428000
6	-2.245748000	-2.438428000	0.736349000
6	-3.024617000	-3.124294000	1.657612000
6	-2.708778000	-3.027557000	3.017533000
6	-1.608622000	-2.279042000	3.441155000
6	-0.822025000	-1.581722000	2.520803000
6	-1.160098000	-1.630394000	1.170329000
6	-1.168634000	1.630034000	-1.169138000
6	-0.840337000	1.581302000	-2.522029000
6	-1.633587000	2.278492000	-3.436757000
6	-2.730656000	3.027036000	-3.005285000
6	-3.036588000	3.123978000	-1.643116000
6	-2.251078000	2.438232000	-0.727429000
6	-2.245747000	2.438428000	0.736349000
6	-3.024617000	3.124294000	1.657612000
6	-2.708777000	3.027556000	3.017533000

6	-1.608622000	2.279042000	3.441156000
6	-0.822025000	1.581722000	2.520802000
6	-1.160099000	1.630394000	1.170329000
6	1.845666000	0.000000000	-0.005133000
6	2.732487000	-1.180369000	-0.005441000
6	2.436372000	-2.540070000	-0.005176000
6	3.491495000	-3.455385000	-0.005600000
6	4.818675000	-3.015589000	-0.006233000
6	5.120457000	-1.651584000	-0.006452000
6	4.074251000	-0.734910000	-0.006049000
6	4.074252000	0.734909000	-0.006049000
6	5.120458000	1.651582000	-0.006452000
6	4.818675000	3.015588000	-0.006233000
6	3.491495000	3.455385000	-0.005601000
6	2.436372000	2.540070000	-0.005176000
6	2.732487000	1.180369000	-0.005441000
1	0.037000000	-1.046483000	-2.857388000
1	-1.386877000	-2.246724000	-4.491836000
1	-3.335493000	-3.559462000	-3.730533000
1	-3.863893000	-3.741265000	-1.310351000
1	-3.854370000	-3.741468000	1.330788000
1	-3.308345000	-3.560066000	3.747083000
1	-1.354309000	-2.247384000	4.494432000
1	0.057663000	-1.046818000	2.849830000
1	0.037000000	1.046483000	-2.857389000
1	-1.386878000	2.246723000	-4.491837000
1	-3.335494000	3.559462000	-3.730533000
1	-3.863892000	3.741267000	-1.310352000
1	-3.854369000	3.741470000	1.330788000
1	-3.308344000	3.560067000	3.747083000
1	-1.354309000	2.247386000	4.494431000
1	0.057663000	1.046818000	2.849829000
1	1.409385000	-2.884669000	-0.004612000
1	3.278589000	-4.518239000	-0.005413000
1	5.623046000	-3.742670000	-0.006538000
1	6.152195000	-1.317776000	-0.006911000
1	6.152195000	1.317775000	-0.006912000
1	5.623047000	3.742668000	-0.006539000
1	3.278592000	4.518239000	-0.005414000
1	1.409385000	2.884669000	-0.004614000

1A(CN:4,12) E(UB3LYP) = -1723.66693183 Ha

6	2.014680000	-0.176187000	-1.165096000
6	2.757767000	-1.290959000	-0.741830000
6	3.798313000	-1.770124000	-1.561968000
6	4.108572000	-1.136787000	-2.759325000
6	3.426181000	0.021331000	-3.151341000
6	2.402278000	0.504452000	-2.352493000
6	0.887938000	0.532508000	-0.561219000
6	1.612478000	1.732989000	-2.442035000
6	0.751674000	1.782772000	-1.314269000

6	1.658448000	2.783748000	-3.346530000
6	0.000000000	2.928704000	-1.061909000
6	0.860127000	3.909241000	-3.113778000
6	0.057076000	3.985715000	-1.974069000
1	-0.581738000	3.021485000	-0.157095000
6	-0.751674000	-1.782772000	-1.314269000
6	0.000000000	-2.928704000	-1.061909000
6	-0.057076000	-3.985715000	-1.974069000
6	-0.860127000	-3.909241000	-3.113778000
6	-1.658448000	-2.783748000	-3.346530000
6	-1.612478000	-1.732989000	-2.442035000
6	-0.887938000	-0.532508000	-0.561219000
1	0.581738000	-3.021485000	-0.157095000
6	-2.402278000	-0.504452000	-2.352493000
6	-2.014680000	0.176187000	-1.165096000
6	-3.426181000	-0.021331000	-3.151341000
6	-2.757767000	1.290959000	-0.741830000
6	-4.108572000	1.136787000	-2.759325000
6	-3.798313000	1.770124000	-1.561968000
6	0.984536000	0.650322000	2.691974000
6	2.114312000	1.403719000	2.395291000
6	2.881162000	1.906761000	3.448872000
6	2.516672000	1.660727000	4.775180000
6	1.378372000	0.908589000	5.077532000
6	0.613811000	0.404652000	4.032483000
6	0.000000000	0.000000000	1.807724000
1	2.398390000	1.601756000	1.368633000
1	3.766610000	2.494509000	3.235631000
1	3.124315000	2.060059000	5.579514000
1	1.100699000	0.724002000	6.109222000
6	-0.613811000	-0.404652000	4.032483000
6	-0.984536000	-0.650322000	2.691974000
6	-1.378372000	-0.908589000	5.077532000
6	-2.114312000	-1.403719000	2.395291000
6	-2.516672000	-1.660727000	4.775180000
1	-1.100699000	-0.724002000	6.109222000
6	-2.881162000	-1.906761000	3.448872000
1	-2.398390000	-1.601756000	1.368633000
1	-3.124315000	-2.060059000	5.579514000
1	-3.766610000	-2.494509000	3.235631000
6	0.000000000	0.000000000	0.471545000
1	-4.369784000	2.629249000	-1.233590000
1	-4.911054000	1.525669000	-3.374738000
1	-3.710449000	-0.542049000	-4.058790000
1	-2.313487000	-2.745517000	-4.209989000
1	-0.887327000	-4.740101000	-3.809381000
1	0.521272000	-4.882490000	-1.783827000
1	4.369784000	-2.629249000	-1.233590000
1	4.911054000	-1.525669000	-3.374738000
1	3.710449000	0.542049000	-4.058790000
1	2.313487000	2.745517000	-4.209989000

1	0.887327000	4.740101000	-3.809381000
1	-0.521272000	4.882490000	-1.783827000
6	-2.581379000	1.897801000	0.539856000
7	-2.490344000	2.409868000	1.572092000
6	2.581379000	-1.897801000	0.539856000
7	2.490344000	-2.409868000	1.572092000

1A(F:4) E(UB3LYP) = -1638.40075822 Ha

6	1.362166000	-1.749934000	-0.771310000
6	1.218243000	-2.042157000	-2.120970000
6	2.143217000	-2.823775000	-2.800393000
6	3.226443000	-3.360528000	-2.102382000
6	3.366828000	-3.154375000	-0.725859000
6	2.432935000	-2.368348000	-0.065279000
6	0.543392000	-0.988008000	0.161267000
9	0.119891000	-1.610649000	-2.792734000
1	1.992449000	-3.019719000	-3.854367000
1	3.947444000	-3.970614000	-2.633567000
1	4.181165000	-3.620227000	-0.183365000
6	2.238312000	-2.083713000	1.356950000
6	1.055200000	-1.307766000	1.493829000
6	2.945199000	-2.497295000	2.477685000
6	0.557509000	-1.012444000	2.761895000
6	2.461489000	-2.158185000	3.745530000
1	3.849821000	-3.086717000	2.375843000
6	1.272804000	-1.437033000	3.883509000
1	-0.382988000	-0.494016000	2.878192000
1	3.001421000	-2.477484000	4.629584000
1	0.893904000	-1.212932000	4.874094000
6	1.428449000	1.457595000	-1.237291000
6	1.519606000	1.104004000	-2.582794000
6	2.533123000	1.666676000	-3.362833000
6	3.438296000	2.578688000	-2.816464000
6	3.327657000	2.973432000	-1.478979000
6	2.321937000	2.423831000	-0.696848000
6	0.490573000	1.053181000	-0.186177000
1	0.801246000	0.433074000	-3.028161000
1	2.609672000	1.396480000	-4.409907000
1	4.219188000	3.000667000	-3.438905000
1	4.009301000	3.709034000	-1.066064000
6	1.906621000	2.711806000	0.676931000
6	0.766916000	1.915083000	0.961141000
6	2.377526000	3.621010000	1.613419000
6	0.071161000	2.081420000	2.156370000
6	1.697613000	3.752179000	2.830153000
1	3.247197000	4.234055000	1.403781000
6	0.548207000	3.002622000	3.091722000
1	-0.845268000	1.535476000	2.338372000
1	2.054111000	4.460917000	3.569089000
1	0.015194000	3.144720000	4.024790000
6	-2.690132000	-1.240819000	0.021057000

6	-2.360645000	-2.565715000	0.290580000
6	-3.391426000	-3.495242000	0.446836000
6	-4.729063000	-3.103520000	0.338343000
6	-5.065087000	-1.774224000	0.071036000
6	-4.042915000	-0.844387000	-0.087205000
6	-1.835048000	-0.056449000	-0.190001000
1	-1.326103000	-2.873445000	0.380859000
1	-3.151208000	-4.531424000	0.655821000
1	-5.514362000	-3.840502000	0.464149000
1	-6.104647000	-1.476753000	-0.011182000
6	-4.081597000	0.597421000	-0.367804000
6	-2.752134000	1.074862000	-0.431940000
6	-5.152286000	1.464317000	-0.559072000
6	-2.494692000	2.418231000	-0.688887000
6	-4.888572000	2.811985000	-0.815201000
1	-6.174074000	1.104258000	-0.511740000
6	-3.574180000	3.283853000	-0.879746000
1	-1.478722000	2.789794000	-0.739590000
1	-5.712381000	3.500506000	-0.966698000
1	-3.390052000	4.333020000	-1.080649000
6	-0.497197000	-0.018668000	-0.166782000

1A(Me:4) E(UB3LYP) = -1578.46165095 Hartrees

6	1.248518000	-1.928586000	-0.626249000
6	0.920920000	-2.401959000	-1.910290000
6	1.826593000	-3.287987000	-2.511768000
6	2.979133000	-3.726895000	-1.859343000
6	3.256013000	-3.324585000	-0.549117000
6	2.386619000	-2.438114000	0.065653000
6	0.542919000	-1.017532000	0.262812000
6	2.344608000	-1.934138000	1.439225000
6	1.186893000	-1.120048000	1.570444000
6	3.164912000	-2.175558000	2.531605000
6	0.833869000	-0.603113000	2.816678000
6	2.823352000	-1.621439000	3.770857000
1	4.051835000	-2.792037000	2.433221000
6	1.664165000	-0.854562000	3.911300000
1	-0.077240000	-0.036741000	2.942909000
1	3.454113000	-1.804341000	4.633323000
1	1.399492000	-0.455807000	4.883954000
6	1.505625000	1.295248000	-1.333849000
6	1.730445000	0.710916000	-2.582875000
6	2.791549000	1.163822000	-3.368984000
6	3.620482000	2.199943000	-2.933795000
6	3.388420000	2.817366000	-1.701082000
6	2.337936000	2.373192000	-0.910818000
6	0.498365000	1.058001000	-0.298991000
1	1.094747000	-0.074622000	-2.956171000
1	2.968253000	0.704771000	-4.335093000
1	4.438361000	2.536308000	-3.560715000
1	4.017255000	3.637189000	-1.371136000

6	1.833930000	2.864624000	0.370154000
6	0.703542000	2.078598000	0.718530000
6	2.228871000	3.921750000	1.178360000
6	-0.043838000	2.383791000	1.856539000
6	1.489779000	4.203549000	2.332877000
1	3.088979000	4.528848000	0.917922000
6	0.358928000	3.449765000	2.661952000
1	-0.939847000	1.822308000	2.090862000
1	1.788493000	5.026627000	2.972264000
1	-0.214524000	3.700894000	3.546875000
6	-2.653423000	-1.203110000	0.262583000
6	-2.319190000	-2.484105000	0.694981000
6	-3.344884000	-3.400213000	0.940388000
6	-4.683638000	-3.037835000	0.765550000
6	-5.025392000	-1.751592000	0.340990000
6	-4.008398000	-0.836655000	0.089979000
6	-1.804269000	-0.048889000	-0.092080000
1	-1.284867000	-2.767254000	0.846427000
1	-3.099226000	-4.401116000	1.276395000
1	-5.465039000	-3.762360000	0.965789000
1	-6.065816000	-1.475321000	0.210114000
6	-4.055374000	0.560285000	-0.361007000
6	-2.728716000	1.039556000	-0.469214000
6	-5.132838000	1.383929000	-0.669365000
6	-2.483762000	2.343230000	-0.893129000
6	-4.880566000	2.692061000	-1.088675000
1	-6.151140000	1.020016000	-0.588142000
6	-3.570047000	3.165616000	-1.200322000
1	-1.472226000	2.718097000	-0.981432000
1	-5.709499000	3.347271000	-1.331992000
1	-3.393604000	4.183352000	-1.529129000
6	-0.462093000	-0.027390000	-0.120968000
1	4.122614000	-3.710626000	-0.024340000
1	3.649024000	-4.413288000	-2.365107000
1	1.607744000	-3.661769000	-3.506546000
6	-0.395778000	-2.105468000	-2.584898000
1	-0.373631000	-2.408213000	-3.633679000
1	-1.199547000	-2.660863000	-2.090209000
1	-0.679802000	-1.054159000	-2.534207000

1A(Me:5) E(UB3LYP) = -1578.46338615 Hartrees

6	-1.046223000	-1.711849000	0.669492000
6	-0.574004000	-1.914844000	1.961502000
6	-1.276921000	-2.751974000	2.839632000
6	-2.433941000	-3.394908000	2.383587000
6	-2.888640000	-3.243154000	1.068366000
6	-2.193553000	-2.408858000	0.206924000
6	-0.523622000	-0.890347000	-0.422465000
6	-2.347756000	-2.126185000	-1.221037000
6	-1.297041000	-1.250182000	-1.610054000
6	-3.237779000	-2.617848000	-2.165004000

6	-1.107135000	-0.941335000	-2.954833000
6	-3.069748000	-2.259000000	-3.507692000
1	-4.040985000	-3.285737000	-1.873414000
6	-2.005386000	-1.444997000	-3.899566000
1	-0.255245000	-0.355508000	-3.270655000
1	-3.757840000	-2.638657000	-4.254545000
1	-1.866611000	-1.209986000	-4.948633000
6	-0.956795000	1.484608000	1.321363000
6	-0.456846000	1.174003000	2.584047000
6	-1.118018000	1.665035000	3.712668000
6	-2.254216000	2.466716000	3.581677000
6	-2.733323000	2.824681000	2.316286000
6	-2.079707000	2.345175000	1.189791000
6	-0.484175000	1.120329000	-0.013914000
1	0.451533000	0.597690000	2.690142000
1	-0.736416000	1.431615000	4.700189000
1	-2.754864000	2.836703000	4.469272000
1	-3.591916000	3.480494000	2.221625000
6	-2.267201000	2.624682000	-0.234760000
6	-1.258422000	1.928065000	-0.955317000
6	-3.153449000	3.463177000	-0.895812000
6	-1.104345000	2.144746000	-2.322951000
6	-3.023624000	3.632292000	-2.278899000
1	-3.924395000	3.995192000	-0.349143000
6	-1.998693000	2.993698000	-2.979905000
1	-0.283005000	1.696391000	-2.863393000
1	-3.708719000	4.285949000	-2.806766000
1	-1.887363000	3.166990000	-4.044182000
6	2.727670000	-1.093309000	-0.518462000
6	2.404862000	-2.421117000	-0.782144000
6	3.441479000	-3.342949000	-0.945698000
6	4.777055000	-2.941649000	-0.844913000
6	5.105713000	-1.610330000	-0.578382000
6	4.078040000	-0.687184000	-0.415238000
6	1.864793000	0.084447000	-0.299591000
1	1.371408000	-2.736701000	-0.857870000
1	3.207402000	-4.381026000	-1.152173000
1	5.566689000	-3.673308000	-0.974643000
1	6.143696000	-1.306233000	-0.500524000
6	4.107492000	0.754024000	-0.129067000
6	2.774966000	1.221641000	-0.059024000
6	5.171979000	1.628250000	0.064532000
6	2.506911000	2.561407000	0.205538000
6	4.898160000	2.972363000	0.328776000
1	6.196521000	1.276764000	0.012385000
6	3.580285000	3.434147000	0.398534000
1	1.487397000	2.922804000	0.262967000
1	5.716985000	3.666372000	0.482223000
1	3.388903000	4.480833000	0.605498000
6	0.526398000	0.113149000	-0.310013000
1	-3.764847000	-3.785632000	0.730063000

1	-2.977394000	-4.045901000	3.060539000
1	0.353143000	-1.458571000	2.281622000
6	-0.795831000	-2.928187000	4.259158000
1	-1.213538000	-3.827028000	4.717874000
1	0.294389000	-2.999959000	4.302847000
1	-1.091383000	-2.072100000	4.875907000

1A(Me:6) E(UB3LYP) = -1578.46296228 Hartrees

6	1.590053000	-1.350852000	-0.610597000
6	1.574071000	-1.601629000	-1.979986000
6	2.682787000	-2.208561000	-2.573538000
6	3.805431000	-2.583610000	-1.825078000
6	3.792745000	-2.381627000	-0.434796000
6	2.697375000	-1.784062000	0.167668000
6	0.590447000	-0.765166000	0.277149000
6	2.368992000	-1.565458000	1.577915000
6	1.064615000	-1.003795000	1.640482000
6	3.056342000	-1.878770000	2.741694000
6	0.436951000	-0.829492000	2.871727000
6	2.436135000	-1.654729000	3.976391000
1	4.052153000	-2.306371000	2.698976000
6	1.134910000	-1.151785000	4.038482000
1	-0.586885000	-0.487563000	2.924765000
1	2.961932000	-1.897019000	4.892989000
1	0.655865000	-1.019749000	5.001887000
6	0.892904000	1.770241000	-1.244112000
6	0.934478000	1.427989000	-2.594391000
6	1.757970000	2.158934000	-3.454290000
6	2.517637000	3.229399000	-2.977688000
6	2.441758000	3.614522000	-1.634349000
6	1.623963000	2.896155000	-0.773771000
6	0.130738000	1.208770000	-0.131251000
1	0.309948000	0.635891000	-2.981795000
1	1.797321000	1.898970000	-4.505979000
1	3.151522000	3.783869000	-3.660389000
1	3.000781000	4.473163000	-1.278633000
6	1.255252000	3.121282000	0.624456000
6	0.304140000	2.130183000	0.989819000
6	1.609906000	4.119510000	1.520990000
6	-0.333121000	2.194478000	2.227236000
6	0.999628000	4.147737000	2.780111000
1	2.335420000	4.877829000	1.247328000
6	0.026680000	3.205452000	3.121554000
1	-1.122048000	1.498602000	2.476939000
1	1.266711000	4.923625000	3.488627000
1	-0.463228000	3.265474000	4.086697000
6	-2.501676000	-1.714255000	-0.078193000
6	-1.914530000	-2.938891000	0.225902000
6	-2.729098000	-4.070554000	0.308939000
6	-4.107202000	-3.976508000	0.092497000
6	-4.701122000	-2.749283000	-0.211698000

6	-3.895310000	-1.618608000	-0.296420000
6	-1.908836000	-0.370498000	-0.230514000
1	-0.847664000	-3.016016000	0.396647000
1	-2.287551000	-5.032019000	0.544863000
1	-4.721762000	-4.867074000	0.162456000
1	-5.770718000	-2.683259000	-0.377684000
6	-4.222768000	-0.216628000	-0.591067000
6	-3.027641000	0.537849000	-0.551949000
6	-5.436961000	0.400608000	-0.873282000
6	-3.046715000	1.908330000	-0.793605000
6	-5.451081000	1.776199000	-1.116008000
1	-6.357444000	-0.171827000	-0.904782000
6	-4.269129000	2.522052000	-1.076634000
1	-2.133844000	2.490449000	-0.761781000
1	-6.389572000	2.272205000	-1.337131000
1	-4.301326000	3.588699000	-1.267033000
6	-0.617377000	-0.042105000	-0.100192000
1	4.634729000	-2.714085000	0.164550000
1	2.669706000	-2.405728000	-3.640361000
1	0.699787000	-1.372468000	-2.573169000
6	5.011559000	-3.194591000	-2.494587000
1	5.472316000	-3.961021000	-1.865797000
1	4.750669000	-3.651866000	-3.451684000
1	5.773781000	-2.432116000	-2.690590000

1A(Me:7) E(UB3LYP) = -1578.46303092 Hartrees

6	1.294578000	-1.361581000	-0.991418000
6	0.890508000	-1.485507000	-2.317460000
6	1.771800000	-2.064076000	-3.230383000
6	3.024342000	-2.514829000	-2.816269000
6	3.436167000	-2.446470000	-1.474727000
6	2.548835000	-1.877100000	-0.557079000
6	0.581939000	-0.809894000	0.155888000
6	2.594649000	-1.750235000	0.906534000
6	1.368778000	-1.159311000	1.331751000
6	3.525311000	-2.155664000	1.857862000
6	1.060150000	-1.063485000	2.685976000
6	3.226958000	-2.005082000	3.218259000
1	4.467590000	-2.599151000	1.567970000
6	2.000888000	-1.483598000	3.629078000
1	0.090130000	-0.707382000	3.002753000
1	3.950934000	-2.323128000	3.960008000
1	1.771083000	-1.412574000	4.686013000
6	0.614814000	1.841740000	-1.224830000
6	0.275682000	1.614043000	-2.557116000
6	0.883679000	2.384487000	-3.551383000
6	1.806636000	3.378833000	-3.220222000
6	2.116290000	3.647754000	-1.881954000
6	1.514157000	2.890175000	-0.887645000
6	0.145646000	1.211430000	0.007394000
1	-0.474671000	0.881165000	-2.817318000

1	0.626892000	2.215227000	-4.590851000
1	2.268938000	3.965023000	-4.006344000
1	2.803331000	4.447996000	-1.629200000
6	1.547468000	3.002341000	0.571237000
6	0.669047000	2.019758000	1.105704000
6	2.188054000	3.904061000	1.408699000
6	0.386182000	2.006037000	2.470228000
6	1.937738000	3.847455000	2.784727000
1	2.858776000	4.654612000	1.004875000
6	1.032893000	2.920288000	3.305696000
1	-0.352746000	1.328022000	2.873156000
1	2.430919000	4.547725000	3.449331000
1	0.819954000	2.916861000	4.368589000
6	-2.565356000	-1.637493000	0.143404000
6	-1.991259000	-2.900955000	0.248025000
6	-2.830213000	-4.016975000	0.289755000
6	-4.219159000	-3.869563000	0.227207000
6	-4.799821000	-2.603482000	0.121274000
6	-3.969804000	-1.488037000	0.079381000
6	-1.946773000	-0.298224000	0.077924000
1	-0.915655000	-3.019873000	0.294809000
1	-2.399118000	-5.008232000	0.370977000
1	-4.852551000	-4.748973000	0.260844000
1	-5.877825000	-2.496343000	0.072685000
6	-4.278714000	-0.055354000	-0.028838000
6	-3.061603000	0.664081000	-0.030269000
6	-5.493838000	0.615205000	-0.122280000
6	-3.059333000	2.052493000	-0.126163000
6	-5.486662000	2.008856000	-0.217398000
1	-6.431148000	0.069864000	-0.122003000
6	-4.282831000	2.719905000	-0.219359000
1	-2.129049000	2.607298000	-0.129950000
1	-6.425521000	2.546235000	-0.291159000
1	-4.298575000	3.801147000	-0.294733000
6	-0.638855000	-0.014017000	0.108646000
1	3.696080000	-2.954961000	-3.546038000
1	1.479554000	-2.172278000	-4.268680000
1	-0.099524000	-1.177805000	-2.622979000
6	4.780460000	-2.992922000	-1.066537000
1	4.671849000	-3.839538000	-0.380845000
1	5.343428000	-3.337644000	-1.935858000
1	5.380357000	-2.233806000	-0.555959000

1A(Me:4,5) E(UB3LYP) = -1617.79126801 Hartrees

6	1.601926000	-1.597848000	-0.067346000
6	1.668653000	-2.298116000	-1.282191000
6	2.862491000	-2.998423000	-1.578460000
6	3.907329000	-3.023636000	-0.651504000
6	3.797556000	-2.416097000	0.603518000
6	2.642488000	-1.718067000	0.899934000
6	0.543099000	-0.771541000	0.511893000

6	2.187678000	-1.096097000	2.144289000
6	0.880967000	-0.587044000	1.922289000
6	2.772021000	-1.008270000	3.399085000
6	0.147930000	-0.046886000	2.976839000
6	2.043013000	-0.431322000	4.446511000
1	3.772155000	-1.389919000	3.573466000
6	0.741490000	0.030426000	4.239526000
1	-0.872778000	0.276691000	2.830022000
1	2.486810000	-0.360074000	5.433072000
1	0.181231000	0.446552000	5.068938000
6	1.364443000	1.358763000	-1.367934000
6	1.765808000	0.663369000	-2.508876000
6	2.848631000	1.138066000	-3.252053000
6	3.520350000	2.303770000	-2.878382000
6	3.099334000	3.035780000	-1.763294000
6	2.024457000	2.571908000	-1.018515000
6	0.289799000	1.120847000	-0.405525000
1	1.232878000	-0.212803000	-2.838659000
1	3.164201000	0.597124000	-4.137139000
1	4.359476000	2.654934000	-3.468077000
1	3.600501000	3.958771000	-1.492563000
6	1.326419000	3.156280000	0.125900000
6	0.251098000	2.291959000	0.463701000
6	1.514123000	4.345682000	0.815858000
6	-0.654896000	2.652126000	1.460376000
6	0.618538000	4.684390000	1.836810000
1	2.332898000	5.010603000	0.563381000
6	-0.460996000	3.852715000	2.146288000
1	-1.513935000	2.029223000	1.672627000
1	0.753544000	5.611690000	2.382125000
1	-1.160071000	4.146603000	2.920864000
6	-2.584446000	-1.463334000	0.240901000
6	-2.126911000	-2.611200000	0.882926000
6	-3.046947000	-3.609901000	1.210648000
6	-4.403483000	-3.459320000	0.907950000
6	-4.869026000	-2.307494000	0.269237000
6	-3.957331000	-1.311222000	-0.063754000
6	-1.861329000	-0.267873000	-0.234649000
1	-1.079346000	-2.728425000	1.131612000
1	-2.705423000	-4.508957000	1.710837000
1	-5.101952000	-4.244532000	1.174870000
1	-5.922733000	-2.195810000	0.038532000
6	-4.141869000	-0.015104000	-0.729177000
6	-2.880315000	0.616915000	-0.833199000
6	-5.285582000	0.603832000	-1.222794000
6	-2.766251000	1.867514000	-1.434944000
6	-5.164947000	1.859044000	-1.823241000
1	-6.254574000	0.123166000	-1.145109000
6	-3.918419000	2.483189000	-1.928950000
1	-1.805276000	2.359084000	-1.516786000
1	-6.047090000	2.355251000	-2.212163000

1	-3.844444000	3.457420000	-2.398497000
6	-0.536300000	-0.059392000	-0.169826000
1	4.597401000	-2.508664000	1.329741000
1	4.813665000	-3.566395000	-0.899059000
6	0.477060000	-2.447532000	-2.196911000
1	0.737319000	-2.293921000	-3.247580000
1	0.083802000	-3.467580000	-2.119625000
1	-0.332967000	-1.768513000	-1.941917000
6	2.999776000	-3.748417000	-2.881488000
1	3.969653000	-4.244014000	-2.949118000
1	2.224250000	-4.513814000	-2.989820000
1	2.906239000	-3.078332000	-3.742769000

1A(Me:4,6) E(UB3LYP) = -1617.79256281 Hartrees

6	1.638203000	-1.429994000	-0.259371000
6	1.569501000	-2.119849000	-1.483427000
6	2.733408000	-2.767882000	-1.917556000
6	3.919175000	-2.788587000	-1.173751000
6	3.942502000	-2.172594000	0.087427000
6	2.815584000	-1.510584000	0.540483000
6	0.637270000	-0.672884000	0.470679000
6	2.526491000	-0.887212000	1.833449000
6	1.180895000	-0.429661000	1.805594000
6	3.293554000	-0.754531000	2.981545000
6	0.602327000	0.108370000	2.954738000
6	2.715598000	-0.180180000	4.119859000
1	4.322195000	-1.097829000	3.003718000
6	1.381846000	0.235209000	4.106781000
1	-0.436215000	0.405324000	2.964610000
1	3.302912000	-0.072535000	5.024650000
1	0.941948000	0.654570000	5.004424000
6	1.021562000	1.649823000	-1.343945000
6	1.535310000	1.023553000	-2.482842000
6	2.488112000	1.687175000	-3.257678000
6	2.923569000	2.971296000	-2.922406000
6	2.395720000	3.624381000	-1.804729000
6	1.450228000	2.972640000	-1.025541000
6	0.030822000	1.235579000	-0.351305000
1	1.203497000	0.042578000	-2.779932000
1	2.889702000	1.196919000	-4.137384000
1	3.663301000	3.468711000	-3.539276000
1	2.716632000	4.629832000	-1.554550000
6	0.699805000	3.426819000	0.143078000
6	-0.174114000	2.375236000	0.529060000
6	0.682641000	4.637719000	0.821957000
6	-1.081486000	2.560537000	1.573412000
6	-0.212984000	4.805747000	1.883804000
1	1.344111000	5.446591000	0.531319000
6	-1.092992000	3.781433000	2.248339000
1	-1.785604000	1.779328000	1.832869000
1	-0.235433000	5.746805000	2.421794000

1	-1.795594000	3.940753000	3.058307000
6	-2.349769000	-1.799177000	0.338556000
6	-1.694954000	-2.864991000	0.951134000
6	-2.424524000	-4.015856000	1.258931000
6	-3.789598000	-4.095719000	0.968474000
6	-4.454100000	-3.026307000	0.363378000
6	-3.731837000	-1.879925000	0.049422000
6	-1.848291000	-0.488060000	-0.118475000
1	-0.641052000	-2.804453000	1.192849000
1	-1.927110000	-4.853292000	1.734715000
1	-4.338901000	-4.996121000	1.219840000
1	-5.513926000	-3.092491000	0.143480000
6	-4.146364000	-0.620037000	-0.580836000
6	-3.014633000	0.223042000	-0.680760000
6	-5.386300000	-0.200018000	-1.050547000
6	-3.126960000	1.484856000	-1.259456000
6	-5.493262000	1.068893000	-1.624040000
1	-6.255572000	-0.844058000	-0.975870000
6	-4.374919000	1.901121000	-1.728432000
1	-2.266637000	2.135994000	-1.343829000
1	-6.452915000	1.411909000	-1.994260000
1	-4.476327000	2.882020000	-2.178568000
6	-0.573285000	-0.066487000	-0.082642000
1	4.834396000	-2.230804000	0.703044000
1	2.705066000	-3.299149000	-2.864176000
6	0.286081000	-2.291049000	-2.257724000
1	0.480258000	-2.702428000	-3.250201000
1	-0.377703000	-2.982776000	-1.728599000
1	-0.274162000	-1.362513000	-2.371552000
6	5.154427000	-3.463101000	-1.715027000
1	5.727583000	-3.944928000	-0.918647000
1	4.905607000	-4.219621000	-2.462604000
1	5.814396000	-2.730812000	-2.193828000

1A(Me:4,7) E(UB3LYP) = -1617.79206689 Hartrees

6	1.493098000	-1.531714000	-0.648640000
6	1.270822000	-2.040402000	-1.940396000
6	2.351270000	-2.686645000	-2.552286000
6	3.571514000	-2.855101000	-1.902541000
6	3.778940000	-2.431639000	-0.580683000
6	2.716461000	-1.780280000	0.047405000
6	0.592868000	-0.814211000	0.236869000
6	2.541648000	-1.317626000	1.431823000
6	1.223176000	-0.790685000	1.550462000
6	3.363572000	-1.366289000	2.553154000
6	0.727990000	-0.383592000	2.787615000
6	2.869769000	-0.919918000	3.786004000
1	4.372754000	-1.749097000	2.494529000
6	1.563129000	-0.446553000	3.904839000
1	-0.293590000	-0.046513000	2.886855000
1	3.508358000	-0.960628000	4.661175000

1	1.189188000	-0.131320000	4.872189000
6	1.058843000	1.670632000	-1.324757000
6	1.417486000	1.162248000	-2.575990000
6	2.370966000	1.836644000	-3.340941000
6	2.958471000	3.018053000	-2.883581000
6	2.585067000	3.558436000	-1.649332000
6	1.640656000	2.894427000	-0.879692000
6	0.109677000	1.215654000	-0.308474000
1	0.965282000	0.266135000	-2.966985000
1	2.652735000	1.436508000	-4.308341000
1	3.695800000	3.526047000	-3.494677000
1	3.022821000	4.488237000	-1.302488000
6	1.027499000	3.254206000	0.397197000
6	0.081850000	2.244834000	0.720478000
6	1.183122000	4.359871000	1.221653000
6	-0.725197000	2.372140000	1.851749000
6	0.387784000	4.466311000	2.368306000
1	1.900804000	5.136240000	0.979929000
6	-0.563685000	3.488254000	2.673636000
1	-1.486659000	1.633225000	2.068234000
1	0.500125000	5.325333000	3.020211000
1	-1.187155000	3.602107000	3.553042000
6	-2.489905000	-1.665108000	0.234153000
6	-1.892475000	-2.850115000	0.656920000
6	-2.701776000	-3.962912000	0.898171000
6	-4.087507000	-3.888914000	0.729512000
6	-4.693524000	-2.700402000	0.315307000
6	-3.892482000	-1.590586000	0.068136000
6	-1.903751000	-0.356030000	-0.114500000
1	-0.821277000	-2.910412000	0.804466000
1	-2.249911000	-4.891998000	1.226353000
1	-4.698408000	-4.762762000	0.926547000
1	-5.769317000	-2.647962000	0.189265000
6	-4.233887000	-0.232014000	-0.372583000
6	-3.038023000	0.516232000	-0.480760000
6	-5.461812000	0.348233000	-0.672608000
6	-3.074915000	1.844817000	-0.896745000
6	-5.492298000	1.682630000	-1.083681000
1	-6.380302000	-0.222502000	-0.591264000
6	-4.311130000	2.421891000	-1.195726000
1	-2.165411000	2.424794000	-0.985174000
1	-6.441523000	2.150200000	-1.320323000
1	-4.354017000	3.455925000	-1.518209000
6	-0.595620000	-0.053400000	-0.148003000
1	4.379511000	-3.358677000	-2.423592000
1	2.226777000	-3.085927000	-3.553504000
6	-0.080931000	-2.033363000	-2.610759000
1	0.002739000	-2.339903000	-3.655369000
1	-0.752685000	-2.735177000	-2.105701000
1	-0.576544000	-1.062723000	-2.575412000
6	5.094138000	-2.697363000	0.105558000

1	4.966945000	-3.369644000	0.960249000
1	5.805713000	-3.160981000	-0.580291000
1	5.541859000	-1.772425000	0.480674000

1A(Me:4,8) E(UB3LYP) = -1617.79209127 Hartrees

6	1.374311000	-1.558458000	-0.920817000
6	1.062172000	-1.964355000	-2.230104000
6	2.073857000	-2.607596000	-2.957595000
6	3.320226000	-2.878246000	-2.398106000
6	3.597189000	-2.555970000	-1.064692000
6	2.621748000	-1.909781000	-0.315952000
6	0.562179000	-0.873116000	0.068305000
6	2.552434000	-1.548189000	1.107142000
6	1.267605000	-0.980672000	1.340309000
6	3.452960000	-1.721526000	2.161738000
6	0.853378000	-0.650579000	2.628483000
6	3.024972000	-1.345261000	3.446417000
6	1.748916000	-0.834847000	3.681173000
1	-0.146248000	-0.284988000	2.811714000
1	3.705784000	-1.473971000	4.281464000
1	1.449734000	-0.584457000	4.692595000
6	1.048696000	1.686928000	-1.357537000
6	1.277413000	1.258327000	-2.667566000
6	2.209594000	1.935432000	-3.456146000
6	2.903072000	3.042292000	-2.962411000
6	2.658832000	3.505946000	-1.666101000
6	1.736271000	2.838685000	-0.873169000
6	0.154217000	1.207218000	-0.303804000
1	0.738239000	0.424825000	-3.086003000
1	2.390413000	1.597207000	-4.470176000
1	3.621970000	3.553740000	-3.592294000
1	3.178150000	4.380507000	-1.289398000
6	1.242561000	3.133901000	0.470426000
6	0.262949000	2.157451000	0.793814000
6	1.526375000	4.163511000	1.356764000
6	-0.450548000	2.244905000	1.989694000
6	0.825049000	4.227801000	2.566264000
1	2.271043000	4.914292000	1.115818000
6	-0.160694000	3.285198000	2.873683000
1	-1.239240000	1.536852000	2.211021000
1	1.037663000	5.027508000	3.266825000
1	-0.710496000	3.367690000	3.804242000
6	-2.556550000	-1.573870000	0.220419000
6	-1.996344000	-2.816127000	0.508004000
6	-2.846269000	-3.902816000	0.727579000
6	-4.234380000	-3.747523000	0.670274000
6	-4.802253000	-2.502128000	0.391210000
6	-3.961134000	-1.417463000	0.166341000
6	-1.925672000	-0.272338000	-0.074311000
1	-0.922245000	-2.940356000	0.569056000
1	-2.423896000	-4.875714000	0.951575000

1	-4.876727000	-4.602691000	0.848366000
1	-5.879795000	-2.387073000	0.350771000
6	-4.257854000	-0.013735000	-0.146929000
6	-3.033387000	0.680353000	-0.290435000
6	-5.470181000	0.648423000	-0.309278000
6	-3.026465000	2.037470000	-0.602979000
6	-5.456267000	2.010474000	-0.617752000
1	-6.410653000	0.119648000	-0.200221000
6	-4.247160000	2.696666000	-0.764227000
1	-2.094962000	2.576814000	-0.716472000
1	-6.392739000	2.541579000	-0.746583000
1	-4.255917000	3.753474000	-1.005350000
6	-0.608612000	-0.032473000	-0.180856000
1	4.552931000	-2.824010000	-0.637768000
1	4.076393000	-3.375690000	-2.995295000
1	1.868022000	-2.922539000	-3.975229000
6	-0.325988000	-1.860181000	-2.812684000
1	-0.311444000	-2.039170000	-3.889724000
1	-0.976963000	-2.612609000	-2.354964000
1	-0.801404000	-0.896948000	-2.628061000
6	4.831868000	-2.297752000	1.964009000
1	5.393855000	-2.297090000	2.899759000
1	4.782109000	-3.330513000	1.604098000
1	5.401908000	-1.722184000	1.228663000

1A(Me:4,9) E(UB3LYP) = -1617.79205567 Hartrees

6	-1.202264000	-1.586418000	1.202684000
6	-0.747890000	-1.904835000	2.495163000
6	-1.646504000	-2.575488000	3.338289000
6	-2.917803000	-2.959583000	2.911659000
6	-3.332690000	-2.721973000	1.597342000
6	-2.471899000	-2.048429000	0.746306000
6	-0.538442000	-0.907872000	0.097974000
6	-2.570640000	-1.758707000	-0.685792000
6	-1.365584000	-1.121348000	-1.085277000
6	-3.555089000	-2.055654000	-1.614103000
6	-1.148276000	-0.844342000	-2.433497000
6	-3.358275000	-1.741210000	-2.969411000
1	-4.475016000	-2.541049000	-1.302653000
6	-2.147965000	-1.152575000	-3.358003000
1	-0.212553000	-0.421139000	-2.768852000
1	-1.978986000	-0.938156000	-4.408070000
6	-1.016581000	1.712249000	1.421222000
6	-1.112878000	1.348668000	2.767058000
6	-1.996344000	2.036941000	3.600365000
6	-2.772752000	3.091495000	3.114795000
6	-2.663669000	3.489298000	1.779128000
6	-1.790377000	2.810155000	0.941270000
6	-0.200615000	1.206825000	0.317063000
1	-0.510207000	0.555409000	3.177169000

1	-2.073951000	1.748979000	4.642694000
1	-3.451754000	3.612734000	3.779919000
1	-3.248728000	4.322679000	1.405379000
6	-1.434119000	3.043994000	-0.456487000
6	-0.450754000	2.084280000	-0.816615000
6	-1.837808000	4.010246000	-1.367858000
6	0.145332000	2.125193000	-2.077690000
6	-1.254108000	4.027069000	-2.639587000
1	-2.585427000	4.748720000	-1.099500000
6	-0.265125000	3.101174000	-2.986523000
1	0.936559000	1.431009000	-2.332734000
1	-1.561068000	4.777026000	-3.359931000
1	0.192599000	3.147423000	-3.968073000
6	2.586121000	-1.482307000	-0.286315000
6	2.063243000	-2.761152000	-0.461944000
6	2.942934000	-3.822117000	-0.690044000
6	4.322722000	-3.605963000	-0.751158000
6	4.852395000	-2.324082000	-0.584961000
6	3.981963000	-1.264450000	-0.352414000
6	1.920545000	-0.193742000	-0.011539000
1	0.994490000	-2.933441000	-0.430516000
1	2.549875000	-4.822980000	-0.827440000
1	4.988230000	-4.442318000	-0.933526000
1	5.923410000	-2.162010000	-0.636271000
6	4.236829000	0.166091000	-0.139382000
6	2.996324000	0.815419000	0.063275000
6	5.426209000	0.886508000	-0.109045000
6	2.950579000	2.186664000	0.302014000
6	5.373058000	2.262189000	0.125987000
1	6.378983000	0.392134000	-0.263348000
6	4.148330000	2.904483000	0.330410000
1	2.006472000	2.691805000	0.460082000
1	6.291142000	2.838482000	0.151829000
1	4.126777000	3.972788000	0.512956000
6	0.606684000	-0.003695000	0.190198000
1	-4.299190000	-3.071125000	1.251719000
1	-3.577184000	-3.476819000	3.599691000
1	-1.329679000	-2.823868000	4.345839000
6	0.674659000	-1.680448000	2.945661000
1	0.765802000	-1.802760000	4.026850000
1	1.334368000	-2.411893000	2.466927000
1	1.063908000	-0.699284000	2.673977000
6	-4.440072000	-2.026601000	-3.981743000
1	-4.033944000	-2.098202000	-4.993226000
1	-4.960547000	-2.961607000	-3.757685000
1	-5.190798000	-1.228270000	-3.984053000

1A(Me:4,10) E(UB3LYP) = -1617.79265396 Hartrees

6	1.220304000	-1.900470000	-0.844623000
6	0.897981000	-2.370247000	-2.129370000
6	1.828424000	-3.217416000	-2.750637000

6	3.000408000	-3.622911000	-2.113425000
6	3.274687000	-3.226762000	-0.799905000
6	2.380960000	-2.379106000	-0.166568000
6	0.495342000	-1.017329000	0.059594000
6	2.333602000	-1.890076000	1.212303000
6	1.147755000	-1.123085000	1.362926000
6	3.165210000	-2.098522000	2.300414000
6	0.782746000	-0.630929000	2.611981000
6	2.810967000	-1.555471000	3.542121000
1	4.075770000	-2.679570000	2.201224000
6	1.625194000	-0.831896000	3.714949000
1	-0.157500000	-0.113398000	2.743160000
1	3.461857000	-1.714466000	4.395491000
6	1.404064000	1.290867000	-1.558883000
6	1.552706000	0.739314000	-2.833362000
6	2.592262000	1.183448000	-3.652935000
6	3.472811000	2.178799000	-3.224254000
6	3.314239000	2.766451000	-1.965413000
6	2.285421000	2.331193000	-1.142384000
6	0.437906000	1.047989000	-0.487238000
1	0.870107000	-0.008710000	-3.201727000
1	2.710729000	0.750807000	-4.639911000
1	4.273092000	2.508646000	-3.876853000
1	3.982087000	3.557195000	-1.641166000
6	1.849486000	2.799929000	0.171882000
6	0.707816000	2.040858000	0.543403000
6	2.315531000	3.814684000	0.996213000
6	0.018110000	2.335943000	1.719516000
6	1.635839000	4.083278000	2.190010000
1	3.186097000	4.399224000	0.719336000
6	0.492371000	3.359781000	2.541381000
1	-0.889391000	1.802137000	1.971676000
1	1.990349000	4.873793000	2.841907000
1	-0.036729000	3.602487000	3.455906000
6	-2.705219000	-1.202206000	0.168021000
6	-2.359732000	-2.493302000	0.559066000
6	-3.379142000	-3.405896000	0.840828000
6	-4.722119000	-3.030394000	0.741325000
6	-5.074509000	-1.734434000	0.357047000
6	-4.064048000	-0.822508000	0.070576000
6	-1.865415000	-0.047691000	-0.206941000
1	-1.321157000	-2.786498000	0.651198000
1	-3.125192000	-4.414697000	1.145706000
1	-5.498259000	-3.752698000	0.968331000
1	-6.117972000	-1.448426000	0.283881000
6	-4.121823000	0.582563000	-0.353299000
6	-2.798191000	1.053395000	-0.520029000
6	-5.205894000	1.420938000	-0.591093000
6	-2.562719000	2.363216000	-0.929914000
6	-4.963096000	2.734744000	-0.998115000
1	-6.222129000	1.064126000	-0.464820000

6	-3.655603000	3.200016000	-1.166648000
1	-1.553119000	2.731138000	-1.060778000
1	-5.797264000	3.401236000	-1.186814000
1	-3.486914000	4.222570000	-1.484426000
6	-0.525969000	-0.032136000	-0.292753000
1	4.159042000	-3.588422000	-0.287475000
1	3.690078000	-4.278811000	-2.632969000
1	1.613413000	-3.586054000	-3.748250000
6	-0.438129000	-2.121126000	-2.785028000
1	-0.385905000	-2.296549000	-3.861800000
1	-1.184758000	-2.807268000	-2.370630000
1	-0.820985000	-1.115632000	-2.614169000
6	1.253393000	-0.249100000	5.056478000
1	0.202130000	-0.437391000	5.292664000
1	1.861610000	-0.667936000	5.860921000
1	1.397355000	0.836831000	5.057085000

1A(Me:4,11) E(UB3LYP) = -1617.78836251 Hartrees

6	1.139416000	-1.602392000	-1.175581000
6	0.774068000	-1.683696000	-2.527466000
6	1.740119000	-2.177260000	-3.420828000
6	2.990259000	-2.616746000	-2.993948000
6	3.311799000	-2.628342000	-1.632130000
6	2.381153000	-2.139226000	-0.731040000
6	0.431304000	-1.080500000	0.000378000
6	2.381063000	-2.138663000	0.732846000
6	1.139296000	-1.601462000	1.176853000
6	3.311623000	-2.627172000	1.634366000
6	0.773860000	-1.681724000	2.528780000
6	2.989991000	-2.614528000	2.996145000
1	4.261621000	-3.024124000	1.294551000
6	1.739838000	-2.174635000	3.422584000
1	3.704617000	-2.983270000	3.723353000
1	1.488064000	-2.235668000	4.476513000
6	1.194472000	1.575328000	-1.169653000
6	0.898229000	1.497850000	-2.530128000
6	1.732150000	2.144081000	-3.445045000
6	2.844880000	2.868593000	-3.012484000
6	3.130296000	2.984494000	-1.647777000
6	2.305314000	2.348090000	-0.731383000
6	0.521440000	1.014057000	-0.000443000
1	0.020949000	0.974264000	-2.876404000
1	1.507624000	2.086311000	-4.504076000
1	3.481944000	3.360446000	-3.738836000
1	3.977851000	3.572800000	-1.313254000
6	2.304849000	2.348848000	0.730261000
6	1.193745000	1.576532000	1.168624000
6	3.129205000	2.986259000	1.646526000
6	0.896621000	1.500441000	2.528978000
6	2.842903000	2.871773000	3.011163000
1	3.976972000	3.574226000	1.311945000

6	1.729926000	2.147658000	3.443761000
1	0.019145000	0.977165000	2.875211000
1	3.479457000	3.364431000	3.737418000
1	1.504724000	2.090999000	4.502709000
6	-2.827387000	-1.046114000	0.000197000
6	-2.601676000	-2.419391000	0.000835000
6	-3.701202000	-3.280808000	0.001069000
6	-5.004384000	-2.774073000	0.000703000
6	-5.236298000	-1.396486000	0.000088000
6	-4.145147000	-0.533598000	-0.000169000
6	-1.881894000	0.086308000	-0.000220000
1	-1.593023000	-2.814927000	0.001169000
1	-3.542520000	-4.353166000	0.001533000
1	-5.844658000	-3.459331000	0.000882000
1	-6.249802000	-1.010883000	-0.000166000
6	-4.070151000	0.934107000	-0.000724000
6	-2.706882000	1.310362000	-0.000752000
6	-5.069178000	1.901853000	-0.001151000
6	-2.343492000	2.653958000	-0.001246000
6	-4.699450000	3.248906000	-0.001637000
1	-6.116321000	1.619966000	-0.001111000
6	-3.351658000	3.620670000	-0.001688000
1	-1.300705000	2.946339000	-0.001289000
1	-5.466104000	4.015621000	-0.001988000
1	-3.085206000	4.671385000	-0.002081000
6	-0.544883000	0.012554000	-0.000166000
1	4.261800000	-3.025015000	-1.291995000
1	3.704953000	-2.986077000	-3.720793000
1	1.488381000	-2.239193000	-4.474712000
6	-0.628836000	-1.438265000	-3.029014000
1	-0.622112000	-1.083910000	-4.062728000
1	-1.191657000	-2.377665000	-3.009067000
1	-1.184759000	-0.728782000	-2.421029000
6	-0.629010000	-1.435831000	3.030172000
1	-1.185180000	-0.727358000	2.421271000
1	-1.191564000	-2.375423000	3.011670000
1	-0.622218000	-1.080001000	4.063386000

1A(Me:4,12) E(UB3LYP) = -1617.78954808 Hartrees

6	0.963040000	-2.117636000	-0.458476000
6	0.407076000	-2.710667000	-1.613244000
6	1.135852000	-3.749207000	-2.211770000
6	2.338557000	-4.220043000	-1.686240000
6	2.856149000	-3.679715000	-0.507024000
6	2.169063000	-2.641268000	0.099900000
6	0.508672000	-1.022744000	0.392270000
6	2.433761000	-1.942433000	1.353940000
6	1.401953000	-0.985123000	1.550076000
6	3.436556000	-2.116163000	2.297619000
6	1.374425000	-0.238650000	2.731026000
6	3.413655000	-1.342506000	3.461992000

1	4.223853000	-2.845215000	2.139293000
6	2.385261000	-0.421405000	3.675983000
1	0.581497000	0.463550000	2.930418000
1	4.190143000	-1.466929000	4.208101000
1	2.366944000	0.159835000	4.590940000
6	1.402088000	0.985174000	-1.550025000
6	1.374626000	0.238764000	-2.731019000
6	2.385561000	0.421518000	-3.675873000
6	3.413984000	1.342555000	-3.461737000
6	3.436826000	2.116137000	-2.297316000
6	2.433935000	1.942408000	-1.353734000
6	0.508716000	1.022770000	-0.392289000
1	0.581710000	-0.463417000	-2.930515000
1	2.367296000	-0.159677000	-4.590859000
1	4.190538000	1.466989000	-4.207776000
1	4.224150000	2.845134000	-2.138871000
6	2.169166000	2.641185000	-0.099674000
6	0.963051000	2.117609000	0.458536000
6	2.856267000	3.679538000	0.507394000
6	0.406973000	2.710627000	1.613247000
6	2.338571000	4.219834000	1.686585000
1	3.771268000	4.068470000	0.075046000
6	1.135752000	3.749074000	2.211927000
1	2.861453000	5.026522000	2.188107000
1	0.734272000	4.214444000	3.105996000
6	-2.695070000	-1.046585000	0.543333000
6	-2.403957000	-2.245925000	1.188657000
6	-3.460505000	-3.057456000	1.608388000
6	-4.786999000	-2.670300000	1.396586000
6	-5.085476000	-1.461715000	0.762746000
6	-4.037577000	-0.652051000	0.338067000
6	-1.807958000	0.000027000	-0.000073000
1	-1.378262000	-2.546319000	1.363350000
1	-3.248522000	-3.995750000	2.108003000
1	-5.592653000	-3.313182000	1.733036000
1	-6.116386000	-1.163158000	0.607862000
6	-4.037555000	0.652100000	-0.338363000
6	-2.695035000	1.046647000	-0.543519000
6	-5.085427000	1.461747000	-0.763142000
6	-2.403880000	2.245992000	-1.188813000
6	-4.786908000	2.670332000	-1.396963000
1	-6.116346000	1.163180000	-0.608343000
6	-3.460400000	3.057507000	-1.608641000
1	-1.378172000	2.546409000	-1.363395000
1	-5.592541000	3.313199000	-1.733493000
1	-3.248384000	3.995805000	-2.108236000
6	-0.465121000	0.000020000	-0.000036000
1	3.771067000	-4.068704000	-0.074545000
1	2.861437000	-5.026810000	-2.187640000
1	0.734463000	-4.214580000	-3.105879000
6	-0.956306000	-2.376992000	-2.166458000

1	-1.088374000	-2.821349000	-3.154852000
1	-1.736787000	-2.776046000	-1.511788000
1	-1.143366000	-1.306527000	-2.243438000
6	-0.956529000	2.377029000	2.166212000
1	-1.736877000	2.776146000	1.511423000
1	-1.143654000	1.306567000	2.243114000
1	-1.088750000	2.821360000	3.154599000

1A(Me:4,19) E(UB3LYP) = -1617.78249616 Hartrees

6	1.205881000	-1.884582000	-0.482334000
6	0.893422000	-2.523553000	-1.690982000
6	1.912673000	-3.276307000	-2.297962000
6	3.156772000	-3.455731000	-1.697552000
6	3.411515000	-2.939553000	-0.421590000
6	2.430317000	-2.176026000	0.188131000
6	0.420864000	-0.990298000	0.371656000
6	2.339514000	-1.635210000	1.546350000
6	1.078418000	-0.993136000	1.677089000
6	3.198346000	-1.724959000	2.631467000
6	0.667494000	-0.499454000	2.913700000
6	2.791966000	-1.196130000	3.863001000
1	4.165227000	-2.206814000	2.534822000
6	1.535062000	-0.605095000	4.004015000
1	-0.317525000	-0.070611000	3.035899000
1	3.452220000	-1.264079000	4.720181000
1	1.225300000	-0.226979000	4.971536000
6	1.402229000	1.155316000	-1.385692000
6	1.379555000	0.758950000	-2.729011000
6	2.542507000	0.977952000	-3.485388000
6	3.655663000	1.629918000	-2.961682000
6	3.624029000	2.149000000	-1.662506000
6	2.496280000	1.927381000	-0.890147000
6	0.420042000	1.015976000	-0.303073000
1	2.553095000	0.659491000	-4.523115000
1	4.532105000	1.778567000	-3.582367000
1	4.457913000	2.724549000	-1.276424000
6	2.129444000	2.441105000	0.430385000
6	0.840197000	1.939513000	0.746045000
6	2.770847000	3.315769000	1.294908000
6	0.186450000	2.348230000	1.905944000
6	2.114929000	3.710627000	2.467900000
1	3.758408000	3.700396000	1.064661000
6	0.832142000	3.243978000	2.762337000
1	-0.816476000	2.000521000	2.121354000
1	2.603376000	4.399099000	3.148285000
1	0.331797000	3.580643000	3.663117000
6	-2.817452000	-1.089477000	0.433145000
6	-2.524623000	-2.358938000	0.923462000
6	-3.580702000	-3.214876000	1.244538000
6	-4.907437000	-2.804769000	1.081866000
6	-5.206843000	-1.530000000	0.595455000

6	-4.159491000	-0.673669000	0.271756000
6	-1.929580000	0.008529000	0.003548000
1	-1.498011000	-2.676701000	1.059644000
1	-3.368959000	-4.206436000	1.628057000
1	-5.712778000	-3.483579000	1.339298000
1	-6.238065000	-1.217273000	0.474075000
6	-4.158481000	0.699688000	-0.250148000
6	-2.815733000	1.115393000	-0.407649000
6	-5.204947000	1.555564000	-0.577669000
6	-2.521536000	2.386190000	-0.893915000
6	-4.904169000	2.830044000	-1.063916000
1	-6.236481000	1.242648000	-0.459417000
6	-3.576982000	3.240800000	-1.220610000
1	-1.494855000	2.709880000	-1.012752000
1	-5.708735000	3.509233000	-1.322764000
1	-3.364074000	4.234305000	-1.598351000
6	-0.590519000	-0.016250000	-0.053240000
1	4.347891000	-3.150424000	0.082652000
1	3.914679000	-4.044306000	-2.202342000
1	1.705139000	-3.764980000	-3.244722000
6	-0.511826000	-2.615348000	-2.231098000
1	-0.524601000	-2.674897000	-3.321147000
1	-0.972933000	-3.533024000	-1.849706000
1	-1.147074000	-1.791655000	-1.918004000
6	0.129959000	0.289364000	-3.427509000
1	0.305091000	-0.619113000	-4.005914000
1	-0.689923000	0.112088000	-2.736223000
1	-0.198932000	1.063912000	-4.128475000

1A(OH:5,10,13,18) E(UB3LYP) = -1840.12921639 Hartrees

6	0.555624000	1.262649000	-1.654743000
6	-0.236367000	2.402919000	-1.630353000
6	0.238306000	3.559889000	-2.253683000
6	1.464923000	3.561600000	-2.921604000
6	2.233755000	2.392317000	-2.994597000
6	1.785305000	1.239570000	-2.370010000
6	0.341849000	-0.045812000	-1.043269000
1	-1.217060000	2.407776000	-1.177942000
1	1.815526000	4.470708000	-3.400542000
1	3.169851000	2.401414000	-3.541867000
6	2.308713000	-0.126835000	-2.315182000
6	1.387930000	-0.914859000	-1.568860000
6	3.427708000	-0.725852000	-2.870500000
6	1.552744000	-2.289196000	-1.458811000
6	3.634285000	-2.101499000	-2.696996000
1	4.145570000	-0.145419000	-3.439333000
6	2.698362000	-2.872781000	-2.005771000
1	0.806947000	-2.919000000	-0.997212000
1	4.517176000	-2.573722000	-3.116745000
6	0.525731000	1.359303000	1.576080000
6	-0.257466000	2.502280000	1.447200000

6	0.190448000	3.691770000	2.031883000
6	1.370277000	3.720787000	2.778455000
6	2.125213000	2.557300000	2.953156000
6	1.713520000	1.376021000	2.352779000
6	0.340703000	0.017034000	1.026012000
1	-1.210343000	2.470678000	0.935836000
1	1.678355000	4.659719000	3.221695000
1	3.028327000	2.589207000	3.552637000
6	2.253611000	0.016189000	2.378369000
6	1.386580000	-0.814112000	1.618542000
6	3.356276000	-0.537109000	3.012430000
6	1.601004000	-2.187631000	1.552690000
6	3.612595000	-1.906049000	2.886308000
1	4.026104000	0.081866000	3.599109000
6	2.742700000	-2.721496000	2.160901000
1	0.887927000	-2.842880000	1.072470000
1	4.479570000	-2.358699000	3.351549000
6	-2.800196000	-0.891543000	-1.161888000
6	-2.515680000	-0.859539000	-2.523764000
6	-4.836374000	-1.414215000	-2.979514000
6	-5.127137000	-1.445462000	-1.613511000
6	-4.106295000	-1.183209000	-0.705933000
6	-1.934539000	-0.656871000	0.011294000
1	-1.517322000	-0.631905000	-2.877095000
6	-3.545034000	-1.124404000	-3.430065000
1	-5.621430000	-1.616070000	-3.699619000
1	-6.131259000	-1.669825000	-1.271099000
6	-4.104614000	-1.139930000	0.763096000
6	-2.797414000	-0.822369000	1.198158000
6	-5.123524000	-1.347900000	1.686722000
6	-2.509592000	-0.710678000	2.555230000
6	-4.829615000	-1.236241000	3.047856000
1	-6.128466000	-1.591732000	1.360378000
6	-3.537163000	-0.921031000	3.477701000
1	-1.510615000	-0.463355000	2.893121000
1	-5.613035000	-1.394977000	3.780416000
1	-3.329833000	-0.837678000	4.538335000
6	-0.630755000	-0.351697000	0.000833000
8	-0.508394000	4.865658000	1.914761000
1	-1.175322000	4.779607000	1.225078000
8	-0.564832000	4.671328000	-2.170202000
1	-0.156580000	5.407632000	-2.637058000
8	2.839166000	-4.227645000	-1.819380000
1	3.647143000	-4.535454000	-2.242834000
8	3.042824000	-4.057901000	2.086555000
1	2.540562000	-4.463666000	1.371331000
1	-3.340293000	-1.103615000	-4.494315000

1B E(UB3LYP) = -1694.00087982 Hartrees

6	0.000000000	1.179971000	2.781083000
6	0.000000000	2.541518000	2.490582000

6	0.000000000	3.455052000	3.546808000
6	0.000000000	3.013381000	4.873371000
6	0.000000000	1.648960000	5.171036000
6	0.000000000	0.734018000	4.122690000
6	0.000000000	0.000000000	1.887523000
1	0.000000000	2.890194000	1.465426000
1	0.000000000	4.518062000	3.334710000
1	0.000000000	3.738772000	5.679215000
1	0.000000000	1.312084000	6.201808000
6	0.000000000	-0.734018000	4.122690000
6	0.000000000	-1.179971000	2.781083000
6	0.000000000	-1.648960000	5.171036000
6	0.000000000	-2.541518000	2.490582000
6	0.000000000	-3.013381000	4.873371000
1	0.000000000	-1.312084000	6.201808000
6	0.000000000	-3.455052000	3.546808000
1	0.000000000	-2.890194000	1.465426000
1	0.000000000	-3.738772000	5.679215000
1	0.000000000	-4.518062000	3.334710000
6	0.000000000	0.000000000	0.552972000
6	0.000000000	-0.820380000	-0.663696000
6	0.672647000	-1.681864000	-3.498459000
6	1.594982000	-1.831329000	-2.381030000
6	2.872951000	-2.343150000	-2.676607000
6	1.290734000	-1.500859000	-1.039702000
6	3.819400000	-2.569357000	-1.688692000
6	2.258133000	-1.738914000	-0.056130000
6	3.504572000	-2.272644000	-0.364559000
1	4.792906000	-2.969656000	-1.948163000
1	2.043365000	-1.478859000	0.970259000
1	4.230193000	-2.435029000	0.424244000
1	3.110856000	-2.575055000	-3.709596000
6	-1.290734000	-1.500859000	-1.039702000
6	-1.594982000	-1.831329000	-2.381030000
6	-2.258133000	-1.738914000	-0.056130000
6	-2.872951000	-2.343150000	-2.676607000
6	-3.504572000	-2.272644000	-0.364559000
1	-2.043365000	-1.478859000	0.970259000
6	-3.819400000	-2.569357000	-1.688692000
1	-3.110856000	-2.575055000	-3.709596000
1	-4.230193000	-2.435029000	0.424244000
1	-4.792906000	-2.969656000	-1.948163000
6	-0.672647000	-1.681864000	-3.498459000
6	0.000000000	0.820380000	-0.663696000
6	0.672647000	1.681864000	-3.498459000
6	1.594982000	1.831329000	-2.381030000
6	2.872951000	2.343150000	-2.676607000
6	1.290734000	1.500859000	-1.039702000
6	3.819400000	2.569357000	-1.688692000
6	2.258133000	1.738914000	-0.056130000
6	3.504572000	2.272644000	-0.364559000

1	4.792906000	2.969656000	-1.948163000
1	2.043365000	1.478859000	0.970259000
1	4.230193000	2.435029000	0.424244000
1	3.110856000	2.575055000	-3.709596000
6	-1.290734000	1.500859000	-1.039702000
6	-1.594982000	1.831329000	-2.381030000
6	-2.258133000	1.738914000	-0.056130000
6	-2.872951000	2.343150000	-2.676607000
6	-3.504572000	2.272644000	-0.364559000
1	-2.043365000	1.478859000	0.970259000
6	-3.819400000	2.569357000	-1.688692000
1	-3.110856000	2.575055000	-3.709596000
1	-4.230193000	2.435029000	0.424244000
1	-4.792906000	2.969656000	-1.948163000
6	-0.672647000	1.681864000	-3.498459000
1	-1.152443000	-1.637925000	-4.472617000
1	1.152443000	-1.637925000	-4.472617000
1	-1.152443000	1.637925000	-4.472617000
1	1.152443000	1.637925000	-4.472617000

1C E(UB3LYP) = -924.338337630 Hartrees

6	-1.181775000	1.580249000	2.573531000
6	-0.733566000	2.681664000	3.213980000
6	0.000000000	0.812409000	2.106425000
6	0.733566000	2.681664000	3.213980000
6	1.181775000	1.580249000	2.573531000
6	-1.181775000	-1.580249000	2.573531000
6	-0.733566000	-2.681664000	3.213980000
6	0.000000000	-0.812409000	2.106425000
6	0.733566000	-2.681664000	3.213980000
6	1.181775000	-1.580249000	2.573531000
6	0.000000000	1.179242000	-1.316977000
6	0.000000000	2.535212000	-1.010010000
6	0.000000000	3.455427000	-2.061071000
6	0.000000000	3.020594000	-3.390508000
6	0.000000000	1.658006000	-3.700281000
6	0.000000000	0.735331000	-2.659056000
6	0.000000000	0.000000000	-0.433321000
1	0.000000000	2.867880000	0.022095000
1	0.000000000	4.517758000	-1.845072000
1	0.000000000	3.751522000	-4.191416000
1	0.000000000	1.330604000	-4.734101000
6	0.000000000	-0.735331000	-2.659056000
6	0.000000000	-1.179242000	-1.316977000
6	0.000000000	-1.658006000	-3.700281000
6	0.000000000	-2.535212000	-1.010010000
6	0.000000000	-3.020594000	-3.390508000
1	0.000000000	-1.330604000	-4.734101000
6	0.000000000	-3.455427000	-2.061071000
1	0.000000000	-2.867880000	0.022095000
1	0.000000000	-3.751522000	-4.191416000

1	0.000000000	-4.517758000	-1.845072000
6	0.000000000	0.000000000	0.895327000
1	-2.204415000	1.288612000	2.389473000
1	-1.349187000	3.450796000	3.661134000
1	1.349187000	3.450796000	3.661134000
1	2.204415000	1.288612000	2.389473000
1	2.204415000	-1.288612000	2.389473000
1	1.349187000	-3.450796000	3.661134000
1	-1.349187000	-3.450796000	3.661134000
1	-2.204415000	-1.288612000	2.389473000

2A E(UB3LYP) = -1078.07894249 Hartrees

6	1.168108000	1.631296000	0.472204000
6	2.523482000	1.574523000	0.792774000
6	3.441817000	2.244234000	-0.019776000
6	3.013184000	2.973925000	-1.130383000
6	1.650376000	3.076242000	-1.433585000
6	0.731558000	2.416892000	-0.630438000
6	0.000000000	1.049397000	1.125748000
1	2.858690000	1.052841000	1.678141000
1	4.497969000	2.204669000	0.221353000
1	3.740999000	3.485390000	-1.750041000
1	1.320621000	3.675442000	-2.275295000
6	-0.731558000	2.416892000	-0.630438000
6	-1.168108000	1.631296000	0.472204000
6	-1.650376000	3.076242000	-1.433585000
6	-2.523482000	1.574523000	0.792774000
6	-3.013184000	2.973925000	-1.130383000
1	-1.320621000	3.675442000	-2.275295000
6	-3.441817000	2.244234000	-0.019776000
1	-2.858690000	1.052841000	1.678141000
1	-3.740999000	3.485390000	-1.750041000
1	-4.497969000	2.204669000	0.221353000
6	1.168108000	-1.631296000	0.472204000
6	2.523482000	-1.574523000	0.792774000
6	3.441817000	-2.244234000	-0.019776000
6	3.013184000	-2.973925000	-1.130383000
6	1.650376000	-3.076242000	-1.433585000
6	0.731558000	-2.416892000	-0.630438000
6	0.000000000	-1.049397000	1.125748000
1	2.858690000	-1.052841000	1.678141000
1	4.497969000	-2.204669000	0.221353000
1	3.740999000	-3.485390000	-1.750041000
1	1.320621000	-3.675442000	-2.275295000
6	-0.731558000	-2.416892000	-0.630438000
6	-1.168108000	-1.631296000	0.472204000
6	-1.650376000	-3.076242000	-1.433585000
6	-2.523482000	-1.574523000	0.792774000
6	-3.013184000	-2.973925000	-1.130383000
1	-1.320621000	-3.675442000	-2.275295000
6	-3.441817000	-2.244234000	-0.019776000

1	-2.858690000	-1.052841000	1.678141000
1	-3.740999000	-3.485390000	-1.750041000
1	-4.497969000	-2.204669000	0.221353000
6	0.000000000	0.000000000	2.146496000
6	0.000000000	0.000000000	3.473047000
1	0.000000000	0.930834000	4.029007000
1	0.000000000	-0.930834000	4.029007000

2A(F:4,12) E(UB3LYP) = -1276.61545343 Hartrees

6	0.698930000	-1.932247000	0.427386000
6	-0.003775000	-3.058323000	0.840216000
6	0.000000000	-4.233791000	0.100871000
6	0.752579000	-4.300683000	-1.072371000
6	1.525522000	-3.212484000	-1.491617000
6	1.505942000	-2.045951000	-0.741189000
6	0.861852000	-0.605576000	1.002705000
9	-0.674021000	-3.048169000	2.021187000
1	0.755004000	-5.219068000	-1.647551000
1	2.141051000	-3.288830000	-2.380267000
6	2.267660000	-0.803965000	-0.865737000
6	1.917824000	0.041321000	0.222233000
6	3.232975000	-0.415934000	-1.784520000
6	2.586258000	1.252323000	0.403344000
6	3.872537000	0.815742000	-1.613856000
1	3.497042000	-1.060478000	-2.615795000
6	3.559913000	1.631133000	-0.523154000
1	2.379070000	1.873872000	1.261568000
1	4.629571000	1.131618000	-2.322509000
1	4.085592000	2.569558000	-0.387839000
6	-1.917824000	-0.041321000	0.222233000
6	-2.586258000	-1.252323000	0.403344000
6	-3.559913000	-1.631133000	-0.523154000
6	-3.872537000	-0.815742000	-1.613856000
6	-3.232975000	0.415934000	-1.784520000
6	-2.267660000	0.803965000	-0.865737000
6	-0.861852000	0.605576000	1.002705000
1	-2.379070000	-1.873872000	1.261568000
1	-4.085592000	-2.569558000	-0.387839000
1	-4.629571000	-1.131618000	-2.322509000
1	-3.497042000	1.060478000	-2.615795000
6	-1.505942000	2.045951000	-0.741189000
6	-0.698930000	1.932247000	0.427386000
6	-1.525522000	3.212484000	-1.491617000
6	0.003775000	3.058323000	0.840216000
6	-0.752579000	4.300683000	-1.072371000
1	-2.141051000	3.288830000	-2.380267000
6	0.000000000	4.233791000	0.100871000
9	0.674021000	3.048169000	2.021187000
1	-0.755004000	5.219068000	-1.647551000
1	0.566945000	5.082387000	0.462023000
6	0.000000000	0.000000000	2.023558000

6	0.000000000	0.000000000	3.346985000
1	0.762763000	-0.533180000	3.902202000
1	-0.762763000	0.533180000	3.902202000
1	-0.566945000	-5.082387000	0.462023000

2A(Me:4) E(UB3LYP) = -1117.40583366 Hartrees

6	1.912683000	0.646349000	0.391765000
6	3.044389000	-0.092803000	0.777211000
6	4.111299000	-0.156188000	-0.133245000
6	4.090187000	0.522832000	-1.349330000
6	3.004086000	1.335677000	-1.690415000
6	1.930948000	1.403613000	-0.818310000
6	0.636942000	0.887741000	1.059703000
1	4.990025000	-0.733775000	0.135137000
1	4.937844000	0.444117000	-2.020801000
1	3.009896000	1.907446000	-2.611576000
6	0.724790000	2.229842000	-0.856182000
6	-0.024643000	1.960532000	0.320001000
6	0.294948000	3.186235000	-1.764242000
6	-1.173090000	2.700252000	0.599229000
6	-0.879432000	3.898421000	-1.493579000
1	0.862769000	3.389406000	-2.665706000
6	-1.596132000	3.665506000	-0.317849000
1	-1.712209000	2.553983000	1.524164000
1	-1.225287000	4.650503000	-2.193552000
1	-2.487900000	4.245543000	-0.109328000
6	-0.135004000	-1.874003000	0.261400000
6	1.067938000	-2.581085000	0.310584000
6	1.320763000	-3.567315000	-0.645268000
6	0.384108000	-3.864215000	-1.637036000
6	-0.841684000	-3.191363000	-1.675313000
6	-1.101846000	-2.209752000	-0.730437000
6	-0.678992000	-0.804683000	1.095777000
1	1.790769000	-2.396734000	1.088334000
1	2.256777000	-4.113193000	-0.610162000
1	0.600671000	-4.632435000	-2.370605000
1	-1.580460000	-3.440704000	-2.429265000
6	-2.298547000	-1.405951000	-0.488716000
6	-2.045639000	-0.585188000	0.643792000
6	-3.537730000	-1.382405000	-1.111747000
6	-3.058074000	0.220825000	1.164178000
6	-4.537622000	-0.546391000	-0.600509000
1	-3.736911000	-2.008398000	-1.974705000
6	-4.303379000	0.236962000	0.532392000
1	-5.510143000	-0.519245000	-1.078958000
1	-5.098261000	0.858435000	0.928799000
6	-0.014664000	0.056302000	2.079413000
6	-0.138663000	0.163385000	3.397155000
1	0.374893000	0.947330000	3.942548000
1	-0.747992000	-0.537524000	3.956641000
6	3.225302000	-0.680338000	2.155985000

1	2.289462000	-0.983285000	2.620962000
1	3.900591000	-1.539351000	2.132265000
1	3.676036000	0.070597000	2.814782000
1	-2.890021000	0.800522000	2.062585000

2A(Me:4,12) E(UB3LYP) = -1156.73149366 Hartrees

6	-2.015899000	-0.687716000	0.500751000
6	-3.146952000	-0.027698000	1.024634000
6	-4.301348000	-0.003252000	0.228539000
6	-4.365415000	-0.636195000	-1.012354000
6	-3.271804000	-1.357485000	-1.497437000
6	-2.112253000	-1.386600000	-0.740408000
6	-0.667491000	-0.869642000	1.023234000
1	-5.181575000	0.506806000	0.605775000
1	-5.281928000	-0.588819000	-1.589765000
1	-3.333304000	-1.887415000	-2.441275000
6	-0.859920000	-2.106520000	-0.955716000
6	-0.001774000	-1.830614000	0.142630000
6	-0.468034000	-2.969688000	-1.968964000
6	1.235951000	-2.472137000	0.224552000
6	0.786051000	-3.583784000	-1.889688000
1	-1.124845000	-3.171215000	-2.808270000
6	1.621431000	-3.342573000	-0.796624000
1	1.887203000	-2.321744000	1.071014000
1	1.105925000	-4.260603000	-2.673728000
1	2.583034000	-3.839487000	-0.735859000
6	0.001757000	1.830557000	0.142816000
6	-1.236034000	2.471955000	0.224712000
6	-1.621532000	3.342474000	-0.796386000
6	-0.786112000	3.583907000	-1.889369000
6	0.468040000	2.969945000	-1.968626000
6	0.859938000	2.106709000	-0.955445000
6	0.667476000	0.869454000	1.023261000
1	-1.887371000	2.321400000	1.071079000
1	-2.583201000	3.839260000	-0.735615000
1	-1.106012000	4.260777000	-2.673355000
1	1.124895000	3.171617000	-2.807863000
6	2.112286000	1.386785000	-0.740232000
6	2.015911000	0.687646000	0.500797000
6	3.271886000	1.357938000	-1.497176000
6	3.146943000	0.027543000	1.024568000
6	4.365513000	0.636575000	-1.012200000
1	3.333433000	1.888104000	-2.440877000
6	4.301396000	0.003316000	0.228510000
1	5.282061000	0.589406000	-1.589572000
1	5.181590000	-0.506873000	0.605643000
6	-0.000023000	-0.000104000	2.005553000
6	-0.000040000	-0.000087000	3.332853000
1	-0.562425000	-0.741319000	3.889681000
1	0.562313000	0.741178000	3.889670000
6	-3.217805000	0.526480000	2.426182000

1	-2.369483000	1.158598000	2.684896000
1	-4.135208000	1.101315000	2.567939000
1	-3.221746000	-0.293332000	3.152445000
6	3.217849000	-0.527048000	2.425958000
1	4.134980000	-1.102411000	2.567312000
1	3.222470000	0.292611000	3.152404000
1	2.369237000	-1.158716000	2.684764000

2A(Me:4,11,12,19) E(UB3LYP) = -1235.37901288 Hartrees

6	1.755999000	-0.178796000	0.114747000
6	2.504022000	-1.354213000	0.271242000
6	3.090004000	-1.902639000	-0.878533000
6	2.987880000	-1.292347000	-2.126986000
6	2.332667000	-0.064468000	-2.261832000
6	1.735603000	0.490885000	-1.142010000
6	0.918433000	0.586650000	1.049157000
6	1.074349000	1.782984000	-0.956783000
6	0.683803000	1.886442000	0.408589000
6	0.868252000	2.825463000	-1.843976000
6	0.201459000	3.104345000	0.913246000
6	0.298775000	4.007636000	-1.360364000
1	1.149000000	2.733162000	-2.887015000
6	0.000000000	4.145909000	-0.006922000
1	0.118352000	4.836427000	-2.035798000
1	-0.380586000	5.095015000	0.356758000
6	-0.683803000	-1.886442000	0.408589000
6	-0.201459000	-3.104345000	0.913246000
6	0.000000000	-4.145909000	-0.006922000
6	-0.298775000	-4.007636000	-1.360364000
6	-0.868252000	-2.825463000	-1.843976000
6	-1.074349000	-1.782984000	-0.956783000
6	-0.918433000	-0.586650000	1.049157000
1	0.380586000	-5.095015000	0.356758000
1	-0.118352000	-4.836427000	-2.035798000
1	-1.149000000	-2.733162000	-2.887015000
6	-1.735603000	-0.490885000	-1.142010000
6	-1.755999000	0.178796000	0.114747000
6	-2.332667000	0.064468000	-2.261832000
6	-2.504022000	1.354213000	0.271242000
6	-2.987880000	1.292347000	-2.126986000
1	-2.295964000	-0.441197000	-3.220337000
6	-3.090004000	1.902639000	-0.878533000
1	-3.452413000	1.755758000	-2.990097000
1	-3.659291000	2.821846000	-0.782412000
6	0.000000000	0.000000000	2.053316000
1	2.295964000	0.441197000	-3.220337000
1	3.452413000	-1.755758000	-2.990097000
1	3.659291000	-2.821846000	-0.782412000
6	2.819959000	-1.954924000	1.617921000
1	2.668854000	-3.036176000	1.621369000
1	3.872323000	-1.769322000	1.859105000

6	-2.819959000	1.954924000	1.617921000
1	-3.872323000	1.769322000	1.859105000
1	-2.221666000	1.519114000	2.414142000
6	-0.047332000	-3.380859000	2.387132000
1	-1.031608000	-3.582490000	2.824470000
1	0.576526000	-4.260415000	2.559765000
6	0.047332000	3.380859000	2.387132000
1	1.031608000	3.582490000	2.824470000
1	-0.576526000	4.260415000	2.559765000
1	2.221666000	-1.519114000	2.414142000
1	0.377738000	-2.543327000	2.933986000
1	-0.377738000	2.543327000	2.933986000
1	-2.668854000	3.036176000	1.621369000
6	0.000000000	0.000000000	3.380465000
1	0.797073000	0.480312000	3.936952000
1	-0.797073000	-0.480312000	3.936952000

2A(Br:4,11,12,19) E(UB3LYP) = -11372.2324152 Hartrees

6	1.697190000	0.003402000	-0.706926000
6	2.565248000	-1.088174000	-0.641455000
6	3.151384000	-1.591422000	-1.801940000
6	2.914725000	-0.976616000	-3.032959000
6	2.143306000	0.184739000	-3.112466000
6	1.555824000	0.674518000	-1.955777000
6	0.850342000	0.673958000	0.283435000
6	0.807995000	1.911301000	-1.713321000
6	0.504790000	1.963637000	-0.321005000
6	0.473391000	2.948815000	-2.565085000
6	0.000000000	3.153505000	0.201521000
6	-0.137178000	4.087234000	-2.026330000
1	0.692093000	2.891355000	-3.624855000
6	-0.343712000	4.205344000	-0.653876000
1	-0.414193000	4.910990000	-2.673938000
1	-0.744047000	5.120535000	-0.237883000
6	-0.504790000	-1.963637000	-0.321005000
6	0.000000000	-3.153505000	0.201521000
6	0.343712000	-4.205344000	-0.653876000
6	0.137178000	-4.087234000	-2.026330000
6	-0.473391000	-2.948815000	-2.565085000
6	-0.807995000	-1.911301000	-1.713321000
6	-0.850342000	-0.673958000	0.283435000
1	0.744047000	-5.120535000	-0.237883000
1	0.414193000	-4.910990000	-2.673938000
1	-0.692093000	-2.891355000	-3.624855000
6	-1.555824000	-0.674518000	-1.955777000
6	-1.697190000	-0.003402000	-0.706926000
6	-2.143306000	-0.184739000	-3.112466000
6	-2.565248000	1.088174000	-0.641455000
6	-2.914725000	0.976616000	-3.032959000
1	-2.014652000	-0.697348000	-4.058807000
6	-3.151384000	1.591422000	-1.801940000

1	-3.378199000	1.383511000	-3.924089000
1	-3.815533000	2.443332000	-1.736681000
6	0.000000000	0.000000000	1.292621000
1	2.014652000	0.697348000	-4.058807000
1	3.378199000	-1.383511000	-3.924089000
1	3.815533000	-2.443332000	-1.736681000
6	0.000000000	0.000000000	2.615165000
1	0.749465000	0.552526000	3.166737000
1	-0.749465000	-0.552526000	3.166737000
35	-3.126335000	1.795631000	1.045668000
35	-0.048083000	3.484021000	2.086997000
35	3.126335000	-1.795631000	1.045668000
35	0.048083000	-3.484021000	2.086997000

2B E(UB3LYP) = -1232.94364852 Hartrees

1	0.000000000	0.925647000	3.857917000
6	0.000000000	0.000000000	3.291635000
1	0.000000000	-0.925647000	3.857917000
6	0.000000000	0.000000000	1.970421000
6	0.000000000	-0.819345000	0.752410000
6	0.672939000	-1.656733000	-2.081079000
6	1.591990000	-1.827304000	-0.963685000
6	2.863197000	-2.356796000	-1.256770000
6	1.282335000	-1.514496000	0.380411000
6	3.791514000	-2.626279000	-0.262370000
6	2.224949000	-1.808044000	1.371740000
6	3.463368000	-2.362285000	1.065979000
1	4.760208000	-3.039751000	-0.519199000
1	1.993643000	-1.571155000	2.401390000
1	4.173675000	-2.567135000	1.858986000
1	3.108471000	-2.572456000	-2.291588000
6	-1.282335000	-1.514496000	0.380411000
6	-1.591990000	-1.827304000	-0.963685000
6	-2.224949000	-1.808044000	1.371740000
6	-2.863197000	-2.356796000	-1.256770000
6	-3.463368000	-2.362285000	1.065979000
1	-1.993643000	-1.571155000	2.401390000
6	-3.791514000	-2.626279000	-0.262370000
1	-3.108471000	-2.572456000	-2.291588000
1	-4.173675000	-2.567135000	1.858986000
1	-4.760208000	-3.039751000	-0.519199000
6	-0.672939000	-1.656733000	-2.081079000
6	0.000000000	0.819345000	0.752410000
6	0.672939000	1.656733000	-2.081079000
6	1.591990000	1.827304000	-0.963685000
6	2.863197000	2.356796000	-1.256770000
6	1.282335000	1.514496000	0.380411000
6	3.791514000	2.626279000	-0.262370000
6	2.224949000	1.808044000	1.371740000
6	3.463368000	2.362285000	1.065979000
1	4.760208000	3.039751000	-0.519199000

1	1.993643000	1.571155000	2.401390000
1	4.173675000	2.567135000	1.858986000
1	3.108471000	2.572456000	-2.291588000
6	-1.282335000	1.514496000	0.380411000
6	-1.591990000	1.827304000	-0.963685000
6	-2.224949000	1.808044000	1.371740000
6	-2.863197000	2.356796000	-1.256770000
6	-3.463368000	2.362285000	1.065979000
1	-1.993643000	1.571155000	2.401390000
6	-3.791514000	2.626279000	-0.262370000
1	-3.108471000	2.572456000	-2.291588000
1	-4.173675000	2.567135000	1.858986000
1	-4.760208000	3.039751000	-0.519199000
6	-0.672939000	1.656733000	-2.081079000
1	-1.153608000	-1.600305000	-3.054218000
1	1.153608000	-1.600305000	-3.054218000
1	-1.153608000	1.600305000	-3.054218000
1	1.153608000	1.600305000	-3.054218000

2C E(UB3LYP) = -463.286259386 Hartrees

6	1.180664000	1.566607000	-0.256888000
6	0.733609000	2.638358000	-0.946201000
6	0.000000000	0.817779000	0.238757000
6	-0.733609000	2.638358000	-0.946201000
6	-1.180664000	1.566607000	-0.256888000
6	1.180664000	-1.566607000	-0.256888000
6	0.733609000	-2.638358000	-0.946201000
6	0.000000000	-0.817779000	0.238757000
6	-0.733609000	-2.638358000	-0.946201000
6	-1.180664000	-1.566607000	-0.256888000
1	0.000000000	0.930115000	3.327073000
6	0.000000000	0.000000000	2.769819000
1	0.000000000	-0.930115000	3.327073000
6	0.000000000	0.000000000	1.451500000
1	2.203555000	1.280304000	-0.065623000
1	1.349485000	3.386309000	-1.427626000
1	-1.349485000	3.386309000	-1.427626000
1	-2.203555000	1.280304000	-0.065623000
1	-2.203555000	-1.280304000	-0.065623000
1	-1.349485000	-3.386309000	-1.427626000
1	1.349485000	-3.386309000	-1.427626000
1	2.203555000	-1.280304000	-0.065623000

2D E(UB3LYP) = -1692.82790653 Hartrees

6	1.163478000	1.607317000	0.966852000
6	2.505744000	1.513158000	1.267709000
6	3.472190000	2.042752000	0.371679000
6	3.039999000	2.694869000	-0.831915000
6	1.644567000	2.827936000	-1.094807000
6	0.731634000	2.304854000	-0.221743000
6	0.000000000	1.091250000	1.656565000

1	2.834237000	1.070677000	2.198342000
1	1.325800000	3.345454000	-1.994121000
6	-0.731634000	2.304854000	-0.221743000
6	-1.163478000	1.607317000	0.966852000
6	-1.644567000	2.827936000	-1.094807000
6	-2.505744000	1.513158000	1.267709000
6	-3.039999000	2.694869000	-0.831915000
1	-1.325800000	3.345454000	-1.994121000
6	-3.472190000	2.042752000	0.371679000
1	-2.834237000	1.070677000	2.198342000
6	1.163478000	-1.607317000	0.966852000
6	2.505744000	-1.513158000	1.267709000
6	3.472190000	-2.042752000	0.371679000
6	3.039999000	-2.694869000	-0.831915000
6	1.644567000	-2.827936000	-1.094807000
6	0.731634000	-2.304854000	-0.221743000
6	0.000000000	-1.091250000	1.656565000
1	2.834237000	-1.070677000	2.198342000
1	1.325800000	-3.345454000	-1.994121000
6	-0.731634000	-2.304854000	-0.221743000
6	-1.163478000	-1.607317000	0.966852000
6	-1.644567000	-2.827936000	-1.094807000
6	-2.505744000	-1.513158000	1.267709000
6	-3.039999000	-2.694869000	-0.831915000
1	-1.325800000	-3.345454000	-1.994121000
6	-3.472190000	-2.042752000	0.371679000
1	-2.834237000	-1.070677000	2.198342000
6	0.000000000	0.000000000	2.644497000
6	4.018632000	3.200345000	-1.720647000
6	5.363149000	3.071311000	-1.446527000
6	5.788538000	2.431793000	-0.261850000
6	4.862054000	1.932853000	0.626008000
6	-4.862054000	1.932853000	0.626008000
6	-5.788538000	2.431793000	-0.261850000
6	-5.363149000	3.071311000	-1.446527000
6	-4.018632000	3.200345000	-1.720647000
6	4.862054000	-1.932853000	0.626008000
6	4.018632000	-3.200345000	-1.720647000
6	-4.018632000	-3.200345000	-1.720647000
6	-4.862054000	-1.932853000	0.626008000
6	-5.363149000	-3.071311000	-1.446527000
6	-5.788538000	-2.431793000	-0.261850000
6	5.363149000	-3.071311000	-1.446527000
6	5.788538000	-2.431793000	-0.261850000
1	3.692238000	3.693433000	-2.630640000
1	6.098706000	3.460992000	-2.141064000
1	6.848102000	2.332664000	-0.054978000
1	5.187449000	1.437836000	1.535018000
1	-3.692238000	3.693433000	-2.630640000
1	-6.098706000	3.460992000	-2.141064000
1	-6.848102000	2.332664000	-0.054978000

1	-5.187449000	1.437836000	1.535018000
1	-5.187449000	-1.437836000	1.535018000
1	-6.848102000	-2.332664000	-0.054978000
1	-6.098706000	-3.460992000	-2.141064000
1	-3.692238000	-3.693433000	-2.630640000
1	5.187449000	-1.437836000	1.535018000
1	6.848102000	-2.332664000	-0.054978000
1	6.098706000	-3.460992000	-2.141064000
1	3.692238000	-3.693433000	-2.630640000
6	0.000000000	0.000000000	3.972160000
1	0.000000000	-0.930832000	4.528420000
1	0.000000000	0.930832000	4.528420000

2E E(UB3LYP) = -2307.55253668 Hartrees

6	1.160402000	1.567853000	1.498679000
6	2.499425000	1.460872000	1.795987000
6	3.478443000	1.832407000	0.827240000
6	3.045886000	2.330814000	-0.462924000
6	1.642612000	2.498871000	-0.713215000
6	0.731520000	2.137947000	0.231684000
6	0.000000000	1.113807000	2.223012000
1	2.822914000	1.113400000	2.768282000
1	1.327622000	2.891761000	-1.674817000
6	-0.731520000	2.137947000	0.231684000
6	-1.160402000	1.567853000	1.498679000
6	-1.642612000	2.498871000	-0.713215000
6	-2.499425000	1.460872000	1.795987000
6	-3.045886000	2.330814000	-0.462924000
1	-1.327622000	2.891761000	-1.674817000
6	-3.478443000	1.832407000	0.827240000
1	-2.822914000	1.113400000	2.768282000
6	1.160402000	-1.567853000	1.498679000
6	2.499425000	-1.460872000	1.795987000
6	3.478443000	-1.832407000	0.827240000
6	3.045886000	-2.330814000	-0.462924000
6	1.642612000	-2.498871000	-0.713215000
6	0.731520000	-2.137947000	0.231684000
6	0.000000000	-1.113807000	2.223012000
1	2.822914000	-1.113400000	2.768282000
1	1.327622000	-2.891761000	-1.674817000
6	-0.731520000	-2.137947000	0.231684000
6	-1.160402000	-1.567853000	1.498679000
6	-1.642612000	-2.498871000	-0.713215000
6	-2.499425000	-1.460872000	1.795987000
6	-3.045886000	-2.330814000	-0.462924000
1	-1.327622000	-2.891761000	-1.674817000
6	-3.478443000	-1.832407000	0.827240000
1	-2.822914000	-1.113400000	2.768282000
6	0.000000000	0.000000000	3.198835000
6	4.004684000	2.621071000	-1.430365000
6	5.377451000	2.438109000	-1.196321000

6	5.808739000	1.961247000	0.091777000
6	4.844454000	1.677809000	1.068703000
6	-4.844454000	1.677809000	1.068703000
6	-5.808739000	1.961247000	0.091777000
6	-5.377451000	2.438109000	-1.196321000
6	-4.004684000	2.621071000	-1.430365000
6	4.844454000	-1.677809000	1.068703000
6	4.004684000	-2.621071000	-1.430365000
6	-4.004684000	-2.621071000	-1.430365000
6	-4.844454000	-1.677809000	1.068703000
6	-5.377451000	-2.438109000	-1.196321000
6	-5.808739000	-1.961247000	0.091777000
6	5.377451000	-2.438109000	-1.196321000
6	5.808739000	-1.961247000	0.091777000
1	3.682334000	2.989277000	-2.399881000
1	5.169577000	1.311747000	2.037288000
1	-3.682334000	2.989277000	-2.399881000
1	-5.169577000	1.311747000	2.037288000
1	-5.169577000	-1.311747000	2.037288000
1	-3.682334000	-2.989277000	-2.399881000
1	5.169577000	-1.311747000	2.037288000
1	3.682334000	-2.989277000	-2.399881000
6	7.203037000	1.778261000	0.321817000
6	6.362028000	2.707352000	-2.190075000
6	-6.362028000	2.707352000	-2.190075000
6	-7.203037000	1.778261000	0.321817000
6	-7.203037000	-1.778261000	0.321817000
6	-6.362028000	-2.707352000	-2.190075000
6	7.203037000	-1.778261000	0.321817000
6	6.362028000	-2.707352000	-2.190075000
6	8.119977000	2.046387000	-0.659550000
6	7.694535000	2.515826000	-1.931607000
6	-7.694535000	2.515826000	-1.931607000
6	-8.119977000	2.046387000	-0.659550000
6	-7.694535000	-2.515826000	-1.931607000
6	-8.119977000	-2.046387000	-0.659550000
6	8.119977000	-2.046387000	-0.659550000
6	7.694535000	-2.515826000	-1.931607000
1	-6.036706000	-3.065600000	-3.161355000
1	-8.432558000	-2.721606000	-2.698672000
1	-9.177154000	-1.895494000	-0.472729000
1	-7.526250000	-1.414972000	1.291559000
1	-6.036706000	3.065600000	-3.161355000
1	-8.432558000	2.721606000	-2.698672000
1	-9.177154000	1.895494000	-0.472729000
1	-7.526250000	1.414972000	1.291559000
1	6.036706000	3.065600000	-3.161355000
1	8.432558000	2.721606000	-2.698672000
1	9.177154000	1.895494000	-0.472729000
1	7.526250000	1.414972000	1.291559000
1	7.526250000	-1.414972000	1.291559000

1	9.177154000	-1.895494000	-0.472729000
1	8.432558000	-2.721606000	-2.698672000
1	6.036706000	-3.065600000	-3.161355000
6	0.000000000	0.000000000	4.526871000
1	0.000000000	0.930638000	5.083493000
1	0.000000000	-0.930638000	5.083493000

3A E(UB3LYP) = -1846.50331444 Hartrees

6	-1.177132000	-1.862065000	1.638932000
6	-2.499511000	-1.427031000	1.590039000
6	-3.472733000	-2.139520000	2.295095000
6	-3.128123000	-3.262724000	3.050159000
6	-1.794730000	-3.676981000	3.145230000
6	-0.821029000	-2.971580000	2.452121000
6	0.035439000	-1.320505000	1.024281000
1	-2.764359000	-0.529708000	1.048693000
1	-4.504786000	-1.809395000	2.264328000
1	-3.897949000	-3.803908000	3.588491000
1	-1.527934000	-4.525167000	3.766250000
6	0.638579000	-3.083476000	2.446954000
6	1.155171000	-2.041291000	1.629727000
6	1.496477000	-3.927857000	3.137427000
6	2.528173000	-1.810752000	1.579528000
6	2.876999000	-3.721417000	3.037137000
1	1.105951000	-4.724401000	3.761333000
6	3.384763000	-2.662774000	2.281265000
1	2.925099000	-0.962381000	1.040344000
1	3.557730000	-4.373097000	3.573064000
1	4.454687000	-2.491865000	2.247584000
6	-1.177132000	-1.862065000	-1.638932000
6	-2.499511000	-1.427031000	-1.590039000
6	-3.472733000	-2.139520000	-2.295095000
6	-3.128123000	-3.262724000	-3.050159000
6	-1.794730000	-3.676981000	-3.145230000
6	-0.821029000	-2.971580000	-2.452121000
6	0.035439000	-1.320505000	-1.024281000
1	-2.764359000	-0.529708000	-1.048693000
1	-4.504786000	-1.809395000	-2.264328000
1	-3.897949000	-3.803908000	-3.588491000
1	-1.527934000	-4.525167000	-3.766250000
6	0.638579000	-3.083476000	-2.446954000
6	1.155171000	-2.041291000	-1.629727000
6	1.496477000	-3.927857000	-3.137427000
6	2.528173000	-1.810752000	-1.579528000
6	2.876999000	-3.721417000	-3.037137000
1	1.105951000	-4.724401000	-3.761333000
6	3.384763000	-2.662774000	-2.281265000
1	2.925099000	-0.962381000	-1.040344000
1	3.557730000	-4.373097000	-3.573064000
1	4.454687000	-2.491865000	-2.247584000
6	0.084030000	1.936329000	1.180656000

6	0.079873000	1.635250000	2.519722000
6	0.074145000	2.685211000	3.476420000
6	0.072243000	4.050555000	3.030521000
6	0.075157000	4.331565000	1.637500000
6	0.080500000	3.299080000	0.733671000
6	0.088689000	1.051831000	0.000000000
1	0.079333000	0.606757000	2.861929000
1	0.072294000	5.365199000	1.306889000
6	0.080500000	3.299080000	-0.733671000
6	0.084030000	1.936329000	-1.180656000
6	0.075157000	4.331565000	-1.637500000
6	0.079873000	1.635250000	-2.519722000
6	0.072243000	4.050555000	-3.030521000
1	0.072294000	5.365199000	-1.306889000
6	0.074145000	2.685211000	-3.476420000
1	0.079333000	0.606757000	-2.861929000
6	0.094294000	-0.287118000	0.000000000
6	0.069349000	2.420197000	4.870298000
6	0.066244000	5.083677000	4.003291000
6	0.062172000	4.791283000	5.347290000
6	0.063655000	3.446423000	5.785775000
6	0.069349000	2.420197000	-4.870298000
6	0.066244000	5.083677000	-4.003291000
6	0.062172000	4.791283000	-5.347290000
6	0.063655000	3.446423000	-5.785775000
1	0.070149000	1.387426000	5.202927000
1	0.060102000	3.228036000	6.847609000
1	0.057640000	5.593082000	6.077066000
1	0.064841000	6.115711000	3.667863000
1	0.064841000	6.115711000	-3.667863000
1	0.057640000	5.593082000	-6.077066000
1	0.060102000	3.228036000	-6.847609000
1	0.070149000	1.387426000	-5.202927000

5F E(UB3LYP) = -1385.51161374 Hartrees

6	0.000000000	1.954397000	-0.050207000
6	0.852972000	2.721149000	0.731351000
6	1.292968000	3.953988000	0.240816000
6	0.873292000	4.412178000	-1.010870000
6	-0.011987000	3.657833000	-1.781189000
6	-0.454798000	2.431925000	-1.288609000
6	-0.590287000	0.566611000	0.233132000
1	1.180700000	2.370311000	1.702738000
1	1.962435000	4.561370000	0.839463000
1	1.225398000	5.369709000	-1.378090000
1	-0.358619000	4.027333000	-2.739971000
6	-1.437340000	1.483865000	-1.820760000
6	-1.591241000	0.425648000	-0.904040000
6	-2.207656000	1.545342000	-2.979512000
6	-2.558572000	-0.546413000	-1.126042000
6	-3.156358000	0.549515000	-3.209864000

1	-2.081846000	2.359929000	-3.684130000
6	-3.338431000	-0.479895000	-2.284185000
1	-2.710403000	-1.351016000	-0.420415000
1	-3.765997000	0.582015000	-4.106017000
1	-4.091934000	-1.238403000	-2.463681000
6	1.591241000	-0.425648000	-0.904040000
6	2.558572000	0.546413000	-1.126042000
6	3.338431000	0.479895000	-2.284185000
6	3.156358000	-0.549515000	-3.209864000
6	2.207656000	-1.545342000	-2.979512000
6	1.437340000	-1.483865000	-1.820760000
6	0.590287000	-0.566611000	0.233132000
1	2.710403000	1.351016000	-0.420415000
1	4.091934000	1.238403000	-2.463681000
1	3.765997000	-0.582015000	-4.106017000
1	2.081846000	-2.359929000	-3.684130000
6	0.454798000	-2.431925000	-1.288609000
6	0.000000000	-1.954397000	-0.050207000
6	0.011987000	-3.657833000	-1.781189000
6	-0.852972000	-2.721149000	0.731351000
6	-0.873292000	-4.412178000	-1.010870000
1	0.358619000	-4.027333000	-2.739971000
6	-1.292968000	-3.953988000	0.240816000
1	-1.180700000	-2.370311000	1.702738000
1	-1.225398000	-5.369709000	-1.378090000
1	-1.962435000	-4.561370000	0.839463000
6	-2.187940000	0.975736000	2.304402000
6	-1.058450000	0.509676000	1.679366000
6	-2.236151000	0.941874000	3.726361000
1	-3.130271000	1.303740000	4.222463000
6	-1.179777000	0.489477000	4.494085000
6	0.000000000	0.000000000	3.869125000
1	-3.020177000	1.379195000	1.739146000
6	0.000000000	0.000000000	2.459682000
6	1.179777000	-0.489477000	4.494085000
6	1.058450000	-0.509676000	1.679366000
6	2.236151000	-0.941874000	3.726361000
1	3.130271000	-1.303740000	4.222463000
6	2.187940000	-0.975736000	2.304402000
1	3.020177000	-1.379195000	1.739146000
1	1.248404000	-0.503466000	5.576655000
1	-1.248404000	0.503466000	5.576655000

6F E(UB3LYP) = -1462.94148756 Hartrees

6	0.408522000	1.950219000	-0.123204000
6	-0.369006000	2.774470000	0.678015000
6	0.136100000	4.022622000	1.053693000
6	1.398748000	4.438022000	0.622199000
6	2.166147000	3.623999000	-0.211454000
6	1.659015000	2.382791000	-0.590321000
6	0.109607000	0.537084000	-0.635324000

1	-1.348095000	2.455020000	1.015056000
1	-0.459079000	4.675334000	1.682447000
1	1.777312000	5.408143000	0.924379000
1	3.133956000	3.959238000	-0.567755000
6	2.184821000	1.372368000	-1.512633000
6	1.251318000	0.322417000	-1.615911000
6	3.350526000	1.371398000	-2.274797000
6	1.463587000	-0.704766000	-2.526629000
6	3.571018000	0.320641000	-3.164958000
1	4.068188000	2.179745000	-2.188221000
6	2.628797000	-0.701255000	-3.298654000
1	0.744703000	-1.504115000	-2.639522000
1	4.472525000	0.304082000	-3.767356000
1	2.801112000	-1.503073000	-4.007712000
6	1.251247000	-0.322022000	1.615803000
6	1.462796000	0.705295000	2.526544000
6	2.627738000	0.702282000	3.298971000
6	3.570401000	-0.319261000	3.165665000
6	3.350631000	-1.370150000	2.275485000
6	2.185199000	-1.371603000	1.512899000
6	0.109883000	-0.537235000	0.634937000
1	0.743563000	1.504370000	2.639137000
1	2.799503000	1.504213000	4.008034000
1	4.471687000	-0.302322000	3.768383000
1	4.068636000	-2.178223000	2.189206000
6	1.660120000	-2.382268000	0.590441000
6	0.409592000	-1.950250000	0.122909000
6	2.167914000	-3.623278000	0.211804000
6	-0.367296000	-2.774873000	-0.678554000
6	1.401150000	-4.437669000	-0.622070000
1	3.135744000	-3.958082000	0.568457000
6	0.138479000	-4.022815000	-1.054021000
1	-1.346398000	-2.455863000	-1.015971000
1	1.780227000	-5.407643000	-0.924079000
1	-0.456204000	-4.675795000	-1.682966000
6	-2.047709000	0.886209000	-2.209252000
6	-1.343722000	0.464778000	-1.097763000
6	-3.486136000	0.850100000	-2.221737000
1	-3.997685000	1.183930000	-3.118646000
6	-4.196679000	0.422200000	-1.113552000
6	-3.458788000	-0.000882000	-0.000218000
1	-1.529137000	1.266125000	-3.082741000
6	-2.080121000	-0.000779000	-0.000299000
6	-4.196475000	-0.423565000	1.113409000
6	-1.343519000	-0.465748000	1.097289000
6	-3.485732000	-0.850867000	2.221691000
1	-3.997119000	-1.184172000	3.118887000
6	-2.047293000	-0.886813000	2.209036000
1	-1.528542000	-1.266064000	3.082709000
6	-5.668467000	-0.270795000	0.745386000
6	-5.668608000	0.269604000	-0.745201000

1	-6.176012000	0.429394000	1.414981000
1	-6.202800000	1.220829000	-0.819752000
1	-6.202639000	-1.222041000	0.819923000
1	-6.176266000	-0.430666000	-1.414638000

7F E(UB3LYP) = -1461.71070737 Hartrees

6	0.791713000	1.446343000	-1.206069000
6	2.163226000	1.478376000	-1.423931000
6	2.653598000	2.072659000	-2.590165000
6	1.782496000	2.632183000	-3.527152000
6	0.406262000	2.630134000	-3.301098000
6	-0.081510000	2.046372000	-2.134561000
6	0.000000000	0.833589000	-0.062923000
1	2.849675000	1.046348000	-0.709312000
1	3.722836000	2.096769000	-2.767370000
1	2.180060000	3.083403000	-4.429446000
1	-0.269888000	3.087008000	-4.015195000
6	-1.446581000	1.989991000	-1.604396000
6	-1.413188000	1.350782000	-0.355549000
6	-2.642676000	2.497132000	-2.107601000
6	-2.557621000	1.262451000	0.424506000
6	-3.800391000	2.378076000	-1.338049000
1	-2.673310000	2.987505000	-3.074291000
6	-3.756745000	1.776832000	-0.077394000
1	-2.528278000	0.797894000	1.403085000
1	-4.738884000	2.770366000	-1.713548000
1	-4.659428000	1.712372000	0.519599000
6	1.413188000	-1.350782000	-0.355549000
6	2.557621000	-1.262451000	0.424506000
6	3.756745000	-1.776832000	-0.077394000
6	3.800391000	-2.378076000	-1.338049000
6	2.642676000	-2.497132000	-2.107601000
6	1.446581000	-1.989991000	-1.604396000
6	0.000000000	-0.833589000	-0.062923000
1	2.528278000	-0.797894000	1.403085000
1	4.659428000	-1.712372000	0.519599000
1	4.738884000	-2.770366000	-1.713548000
1	2.673310000	-2.987505000	-3.074291000
6	0.081510000	-2.046372000	-2.134561000
6	-0.791713000	-1.446343000	-1.206069000
6	-0.406262000	-2.630134000	-3.301098000
6	-2.163226000	-1.478376000	-1.423931000
6	-1.782496000	-2.632183000	-3.527152000
1	0.269888000	-3.087008000	-4.015195000
6	-2.653598000	-2.072659000	-2.590165000
1	-2.849675000	-1.046348000	-0.709312000
1	-2.180060000	-3.083403000	-4.429446000
1	-3.722836000	-2.096769000	-2.767370000
6	0.747965000	2.260481000	2.103595000
6	0.352701000	1.141544000	1.388173000
6	0.774653000	2.242889000	3.542137000

1	1.093117000	3.145513000	4.053845000
6	0.384147000	1.112385000	4.253606000
6	0.000000000	0.000000000	3.485882000
1	1.024462000	3.175671000	1.592263000
6	0.000000000	0.000000000	2.123787000
6	-0.384147000	-1.112385000	4.253606000
6	-0.352701000	-1.141544000	1.388173000
6	-0.774653000	-2.242889000	3.542137000
1	-1.093117000	-3.145513000	4.053845000
6	-0.747965000	-2.260481000	2.103595000
1	-1.024462000	-3.175671000	1.592263000
6	-0.226236000	-0.647885000	5.646713000
6	0.226236000	0.647885000	5.646713000
1	-0.433166000	-1.233379000	6.532323000
1	0.433166000	1.233379000	6.532323000

8F E(UB3LYP) = -1539.21527635 Hartrees

6	1.432711000	1.347881000	-0.229060000
6	2.501212000	1.271871000	0.655959000
6	3.733424000	1.814188000	0.279995000
6	3.891864000	2.434460000	-0.961989000
6	2.814752000	2.536053000	-1.841310000
6	1.586407000	1.995459000	-1.466238000
6	0.000849000	0.798542000	-0.087724000
1	2.394543000	0.796122000	1.621477000
1	4.573685000	1.753772000	0.962464000
1	4.855425000	2.848011000	-1.238134000
1	2.931403000	3.029167000	-2.800020000
6	0.303680000	1.982466000	-2.170274000
6	-0.649275000	1.334185000	-1.366932000
6	-0.046581000	2.512885000	-3.410419000
6	-1.971299000	1.250212000	-1.787522000
6	-1.368699000	2.404168000	-3.838397000
1	0.693964000	3.006766000	-4.029692000
6	-2.324427000	1.785540000	-3.029054000
1	-2.723600000	0.780382000	-1.168367000
1	-1.659605000	2.810255000	-4.800833000
1	-3.352925000	1.718557000	-3.365326000
6	0.649275000	-1.334185000	-1.366932000
6	1.971299000	-1.250212000	-1.787522000
6	2.324427000	-1.785540000	-3.029054000
6	1.368699000	-2.404168000	-3.838397000
6	0.046581000	-2.512885000	-3.410419000
6	-0.303680000	-1.982466000	-2.170274000
6	-0.000849000	-0.798542000	-0.087724000
1	2.723600000	-0.780382000	-1.168367000
1	3.352925000	-1.718557000	-3.365326000
1	1.659605000	-2.810255000	-4.800833000
1	-0.693964000	-3.006766000	-4.029692000
6	-1.586407000	-1.995459000	-1.466238000
6	-1.432711000	-1.347881000	-0.229060000

6	-2.814752000	-2.536053000	-1.841310000
6	-2.501212000	-1.271871000	0.655959000
6	-3.891864000	-2.434460000	-0.961989000
1	-2.931403000	-3.029167000	-2.800020000
6	-3.733424000	-1.814188000	0.279995000
1	-2.394543000	-0.796122000	1.621477000
1	-4.855425000	-2.848011000	-1.238134000
1	-4.573685000	-1.753772000	0.962464000
6	1.432711000	-2.446251000	3.652711000
6	1.725705000	-3.078768000	2.463926000
6	0.724852000	-1.224543000	3.654782000
6	1.297590000	-2.526520000	1.244334000
6	0.339484000	-0.639197000	2.418073000
6	0.606648000	-1.328021000	1.208081000
6	0.358771000	-0.576917000	4.880779000
6	-0.358771000	0.576917000	4.880779000
6	-0.724852000	1.224543000	3.654782000
6	-0.339484000	0.639197000	2.418073000
1	1.733559000	-2.882532000	4.599335000
1	2.271172000	-4.015714000	2.463515000
1	1.499185000	-3.054302000	0.320886000
1	0.650934000	-1.042267000	5.816375000
1	-0.650934000	1.042267000	5.816375000
6	-1.432711000	2.446251000	3.652711000
6	-0.606648000	1.328021000	1.208081000
6	-1.297590000	2.526520000	1.244334000
6	-1.725705000	3.078768000	2.463926000
1	-1.733559000	2.882532000	4.599335000
1	-1.499185000	3.054302000	0.320886000
1	-2.271172000	4.015714000	2.463515000

9F E(UB3LYP) = -1385.46967208 Hartrees

1	0.454551000	2.441713000	5.075733000
6	0.256483000	1.374786000	5.075792000
6	0.129433000	0.694919000	6.263943000
6	-0.129431000	-0.694918000	6.263943000
6	-0.256481000	-1.374785000	5.075792000
6	-0.132321000	-0.708915000	3.828104000
6	0.132325000	0.708916000	3.828104000
6	0.254509000	1.420126000	2.595800000
6	0.118984000	0.693368000	1.447373000
6	-0.118979000	-0.693368000	1.447373000
6	-0.254506000	-1.420125000	2.595800000
1	0.227623000	1.223326000	7.205501000
1	-0.227622000	-1.223324000	7.205501000
1	-0.454548000	-2.441712000	5.075733000
1	-0.437915000	-2.489183000	2.601966000
1	0.437919000	2.489184000	2.601966000
6	1.142732000	1.466933000	-0.906477000
6	2.519942000	1.456884000	-0.740056000
6	3.328549000	2.041729000	-1.719941000

6	2.762188000	2.631839000	-2.850797000
6	1.374944000	2.685663000	-3.002570000
6	0.570348000	2.118090000	-2.019771000
6	0.050823000	0.866553000	-0.056224000
1	2.968577000	1.016046000	0.139302000
1	4.405706000	2.036945000	-1.597860000
1	3.403821000	3.073248000	-3.605220000
1	0.934617000	3.182909000	-3.859839000
6	-0.884719000	2.162237000	-1.832788000
6	-1.188712000	1.530253000	-0.612667000
6	-1.882710000	2.792984000	-2.569273000
6	-2.467498000	1.601414000	-0.083448000
6	-3.182021000	2.820404000	-2.056498000
1	-1.653941000	3.279472000	-3.511106000
6	-3.468199000	2.249241000	-0.815255000
1	-2.685770000	1.173549000	0.888219000
1	-3.969967000	3.313259000	-2.614935000
1	-4.473047000	2.312004000	-0.413088000
6	1.188714000	-1.530259000	-0.612666000
6	2.467498000	-1.601431000	-0.083444000
6	3.468196000	-2.249264000	-0.815250000
6	3.182016000	-2.820423000	-2.056493000
6	1.882707000	-2.792995000	-2.569271000
6	0.884719000	-2.162241000	-1.832787000
6	-0.050818000	-0.866552000	-0.056224000
1	2.685771000	-1.173570000	0.888225000
1	4.473042000	-2.312035000	-0.413080000
1	3.969960000	-3.313284000	-2.614930000
1	1.653935000	-3.279481000	-3.511104000
6	-0.570348000	-2.118087000	-2.019771000
6	-1.142729000	-1.466927000	-0.906478000
6	-1.374947000	-2.685654000	-3.002571000
6	-2.519940000	-1.456866000	-0.740059000
6	-2.762190000	-2.631822000	-2.850800000
1	-0.934622000	-3.182904000	-3.859839000
6	-3.328549000	-2.041706000	-1.719944000
1	-2.968573000	-1.016023000	0.139297000
1	-3.403826000	-3.073226000	-3.605223000
1	-4.405706000	-2.036915000	-1.597865000

10A E(UB3LYP) = -1262.60770256 Hartrees

6	-0.942954000	-0.341979000	1.745354000
6	-2.320120000	-0.545214000	1.669418000
6	-2.880383000	-1.624143000	2.356833000
6	-2.078718000	-2.484863000	3.108510000
6	-0.696562000	-2.277714000	3.203738000
6	-0.131758000	-1.207538000	2.528724000
6	-0.078916000	0.647483000	1.106518000
1	-2.954610000	0.137043000	1.121126000
1	-3.950865000	-1.786583000	2.314463000
1	-2.533031000	-3.315685000	3.635901000

1	-0.083459000	-2.942815000	3.801564000
6	1.242963000	-0.706444000	2.474777000
6	1.258750000	0.445089000	1.637340000
6	2.391724000	-1.113677000	3.132282000
6	2.411773000	1.224723000	1.549861000
6	3.563546000	-0.359830000	2.981526000
1	2.383455000	-1.989391000	3.771485000
6	3.566775000	0.805899000	2.215632000
1	2.404686000	2.166621000	1.021582000
1	4.468237000	-0.668216000	3.492764000
1	4.468164000	1.403802000	2.149892000
6	-0.942954000	-0.341979000	-1.745354000
6	-2.320120000	-0.545214000	-1.669418000
6	-2.880383000	-1.624143000	-2.356833000
6	-2.078718000	-2.484863000	-3.108510000
6	-0.696562000	-2.277714000	-3.203738000
6	-0.131758000	-1.207538000	-2.528724000
6	-0.078916000	0.647483000	-1.106518000
1	-2.954610000	0.137043000	-1.121126000
1	-3.950865000	-1.786583000	-2.314463000
1	-2.533031000	-3.315685000	-3.635901000
1	-0.083459000	-2.942815000	-3.801564000
6	1.242963000	-0.706444000	-2.474777000
6	1.258750000	0.445089000	-1.637340000
6	2.391724000	-1.113677000	-3.132282000
6	2.411773000	1.224723000	-1.549861000
6	3.563546000	-0.359830000	-2.981526000
1	2.383455000	-1.989391000	-3.771485000
6	3.566775000	0.805899000	-2.215632000
1	2.404686000	2.166621000	-1.021582000
1	4.468237000	-0.668216000	-3.492764000
1	4.468164000	1.403802000	-2.149892000
6	-0.534635000	1.468130000	0.000000000
6	-1.527568000	2.400624000	0.000000000
6	-2.063955000	2.907099000	1.222360000
7	-2.495565000	3.310802000	2.215226000
6	-2.063955000	2.907099000	-1.222360000
7	-2.495565000	3.310802000	-2.215226000

10A(Me:4) E(UB3LYP) = -1301.93694293 Hartrees

6	-2.079450000	-0.192793000	0.140324000
6	-2.928314000	0.482963000	1.031903000
6	-4.120231000	1.004834000	0.503970000
6	-4.481463000	0.813571000	-0.827133000
6	-3.683676000	0.044845000	-1.686153000
6	-2.496145000	-0.466360000	-1.197385000
6	-0.811650000	-0.858141000	0.362484000
1	-4.790217000	1.546740000	1.163015000
1	-5.412325000	1.232903000	-1.191800000
1	-4.004095000	-0.157438000	-2.701631000
6	-1.528202000	-1.392683000	-1.791738000

6	-0.544314000	-1.685757000	-0.809040000
6	-1.469758000	-1.990386000	-3.040138000
6	0.473367000	-2.599242000	-1.077011000
6	-0.434841000	-2.896350000	-3.309087000
1	-2.212037000	-1.766960000	-3.798322000
6	0.519088000	-3.201493000	-2.336708000
1	-0.381630000	-3.374830000	-4.280171000
1	1.299388000	-3.920411000	-2.556835000
6	0.442476000	1.882332000	0.424189000
6	-0.604568000	2.668373000	0.909309000
6	-0.771029000	3.964977000	0.417587000
6	0.106437000	4.490972000	-0.530749000
6	1.190110000	3.733191000	-0.988356000
6	1.360331000	2.443781000	-0.509151000
6	0.890336000	0.530528000	0.750069000
1	-1.266149000	2.307170000	1.677683000
1	-1.587972000	4.572224000	0.789789000
1	-0.038309000	5.500410000	-0.898032000
1	1.892006000	4.154284000	-1.699502000
6	2.420306000	1.468133000	-0.753280000
6	2.153884000	0.323794000	0.045842000
6	3.547877000	1.518296000	-1.558486000
6	3.032647000	-0.760979000	0.028859000
6	4.419993000	0.423777000	-1.571547000
1	3.755245000	2.390909000	-2.167734000
6	4.166254000	-0.700459000	-0.782725000
1	5.306682000	0.452137000	-2.194401000
1	4.860768000	-1.532166000	-0.792489000
6	0.201774000	-0.583737000	1.370338000
6	0.646566000	-1.425799000	2.344297000
6	-0.056004000	-2.636480000	2.625909000
7	-0.639627000	-3.608375000	2.850007000
6	1.790825000	-1.119209000	3.140539000
7	2.713895000	-0.867876000	3.788362000
6	-2.694692000	0.524826000	2.521980000
1	-1.642255000	0.466480000	2.797669000
1	-3.118315000	1.428556000	2.966636000
1	-3.192001000	-0.332941000	2.987448000
1	2.866144000	-1.626517000	0.655497000
1	1.197591000	-2.862733000	-0.318889000

10D E(UB3LYP) = -1877.35823311 Hartrees

6	-1.091604000	-1.709114000	0.553955000
6	-2.451208000	-1.610539000	0.758234000
6	-3.349369000	-2.168759000	-0.188122000
6	-2.832109000	-2.841298000	-1.345919000
6	-1.421646000	-2.950645000	-1.521760000
6	-0.573017000	-2.402905000	-0.600117000
6	0.025477000	-1.127199000	1.280660000
1	-2.851281000	-1.140377000	1.646260000
1	-1.038572000	-3.463092000	-2.398374000

6	0.887208000	-2.348047000	-0.529212000
6	1.236526000	-1.599694000	0.655511000
6	1.859911000	-2.874054000	-1.332013000
6	2.555530000	-1.488556000	1.043863000
6	3.235220000	-2.701949000	-0.992964000
1	1.604974000	-3.431305000	-2.227602000
6	3.583219000	-2.020665000	0.221148000
1	2.819238000	-1.047077000	1.994506000
6	-1.091859000	1.709016000	0.554038000
6	-2.451439000	1.610349000	0.758428000
6	-3.349712000	2.168511000	-0.187857000
6	-2.832588000	2.841100000	-1.345687000
6	-1.422146000	2.950551000	-1.521633000
6	-0.573408000	2.402862000	-0.600061000
6	0.025317000	1.127165000	1.280645000
1	-2.851408000	1.140150000	1.646481000
1	-1.039175000	3.463040000	-2.398268000
6	0.886828000	2.348116000	-0.529258000
6	1.236284000	1.599771000	0.655429000
6	1.859437000	2.874213000	-1.332113000
6	2.555321000	1.488723000	1.043692000
6	3.234782000	2.702212000	-0.993155000
1	1.604398000	3.431462000	-2.227675000
6	3.582914000	2.020934000	0.220923000
1	2.819126000	1.047237000	1.994306000
6	-0.168749000	-0.000026000	2.181422000
6	-3.745047000	-3.379957000	-2.283464000
6	-5.105364000	-3.263060000	-2.096619000
6	-5.614352000	-2.602923000	-0.956734000
6	-4.753805000	-2.070825000	-0.023593000
6	4.951434000	-1.888931000	0.564169000
6	5.938632000	-2.390058000	-0.254406000
6	5.596529000	-3.054301000	-1.451786000
6	4.273810000	-3.208944000	-1.808483000
6	-4.754129000	2.070472000	-0.023225000
6	-3.745635000	3.379705000	-2.283157000
6	4.273280000	3.209305000	-1.808731000
6	4.951162000	1.889307000	0.563856000
6	5.596033000	3.054763000	-1.452120000
6	5.938267000	2.390530000	-0.254772000
6	-5.105930000	3.262706000	-2.096210000
6	-5.614784000	2.602518000	-0.956295000
1	-3.356006000	-3.889355000	-3.158958000
1	-5.790495000	-3.680098000	-2.825845000
1	-6.686063000	-2.517549000	-0.818922000
1	-5.141809000	-1.565086000	0.854214000
1	4.013038000	-3.725465000	-2.726340000
1	6.379472000	-3.446547000	-2.090699000
1	6.981409000	-2.275611000	0.018239000
1	5.211706000	-1.377542000	1.484721000
1	5.211534000	1.377929000	1.484386000

1	6.981071000	2.276166000	0.017806000
1	6.378903000	3.447086000	-2.091075000
1	4.012407000	3.725820000	-2.726562000
1	-5.142031000	1.564690000	0.854603000
1	-6.686479000	2.517063000	-0.818405000
1	-5.791146000	3.679702000	-2.825381000
1	-3.356697000	3.889142000	-3.158673000
6	-0.864478000	-0.000061000	3.355244000
6	-1.239654000	1.220730000	3.992865000
7	-1.545593000	2.211220000	4.503681000
6	-1.239456000	-1.220889000	3.992915000
7	-1.545251000	-2.211409000	4.503759000

11A E(UB3LYP) = -1156.74702377 Hartrees

6	-0.763830000	1.857738000	0.120000000
6	0.000000000	2.976224000	0.447876000
6	-0.089872000	4.118785000	-0.351189000
6	-0.942747000	4.151102000	-1.456529000
6	-1.750072000	3.050430000	-1.766167000
6	-1.669072000	1.913489000	-0.975022000
6	-0.881023000	0.551934000	0.765730000
1	0.626560000	2.974606000	1.328788000
1	0.502064000	4.992875000	-0.104417000
1	-0.997358000	5.045764000	-2.066422000
1	-2.439076000	3.095411000	-2.602593000
6	-2.444724000	0.672463000	-0.979338000
6	-2.001773000	-0.122370000	0.113425000
6	-3.499099000	0.251200000	-1.776744000
6	-2.673896000	-1.300721000	0.432489000
6	-4.135919000	-0.958354000	-1.475855000
1	-3.838135000	0.855273000	-2.611313000
6	-3.737221000	-1.716404000	-0.372915000
1	-2.401770000	-1.868250000	1.311268000
1	-4.961712000	-1.298132000	-2.090656000
1	-4.264338000	-2.632814000	-0.132801000
6	2.001773000	0.122370000	0.113425000
6	2.673896000	1.300721000	0.432489000
6	3.737221000	1.716404000	-0.372915000
6	4.135919000	0.958354000	-1.475855000
6	3.499099000	-0.251200000	-1.776744000
6	2.444724000	-0.672463000	-0.979338000
6	0.881023000	-0.551934000	0.765730000
1	2.401770000	1.868250000	1.311268000
1	4.264338000	2.632814000	-0.132801000
1	4.961712000	1.298132000	-2.090656000
1	3.838135000	-0.855273000	-2.611313000
6	1.669072000	-1.913489000	-0.975022000
6	0.763830000	-1.857738000	0.120000000
6	1.750072000	-3.050430000	-1.766167000
6	0.000000000	-2.976224000	0.447876000
6	0.942747000	-4.151102000	-1.456529000

1	2.439076000	-3.095411000	-2.602593000
6	0.089872000	-4.118785000	-0.351189000
1	-0.626560000	-2.974606000	1.328788000
1	0.997358000	-5.045764000	-2.066422000
1	-0.502064000	-4.992875000	-0.104417000
6	0.000000000	0.000000000	1.793705000
6	0.000000000	0.000000000	3.127363000
6	1.087715000	-0.679791000	3.911179000
1	1.829106000	-1.141335000	3.256620000
1	1.598046000	0.037087000	4.564435000
1	0.667312000	-1.454715000	4.562245000
6	-1.087715000	0.679791000	3.911179000
1	-1.829106000	1.141335000	3.256620000
1	-1.598046000	-0.037087000	4.564435000
1	-0.667312000	1.454715000	4.562245000

Ref. 40 in the main text:

Gaussian 16, Revision A.03, Frisch, M. J.; Trucks, G. W.; Schlegel, H. B.; Scuseria, G. E.; Robb, M. A.; Cheeseman, J. R.; Scalmani, G.; Barone, V.; Petersson, G. A.; Nakatsuji, H.; Li, X.; Caricato, M.; Marenich, A. V.; Bloino, J.; Janesko, B. G.; Gomperts, R.; Mennucci, B.; Hratchian, H. P.; Ortiz, J. V.; Izmaylov, A. F.; Sonnenberg, J. L.; Williams-Young, D.; Ding, F.; Lipparini, F.; Egidi, F.; Goings, J.; Peng, B.; Petrone, A.; Henderson, T.; Ranasinghe, D.; Zakrzewski, V. G.; Gao, J.; Rega, N.; Zheng, G.; Liang, W.; Hada, M.; Ehara, M.; Toyota, K.; Fukuda, R.; Hasegawa, J.; Ishida, M.; Nakajima, T.; Honda, Y.; Kitao, O.; Nakai, H.; Vreven, T.; Throssell, K.; Montgomery, J. A., Jr.; Peralta, J. E.; Ogliaro, F.; Bearpark, M. J.; Heyd, J. J.; Brothers, E. N.; Kudin, K. N.; Staroverov, V. N.; Keith, T. A.; Kobayashi, R.; Normand, J.; Raghavachari, K.; Rendell, A. P.; Burant, J. C.; Iyengar, S. S.; Tomasi, J.; Cossi, M.; Millam, J. M.; Klene, M.; Adamo, C.; Cammi, R.; Ochterski, J. W.; Martin, R. L.; Morokuma, K.; Farkas, O.; Foresman, J. B.; Fox, D. J. Gaussian, Inc., Wallingford CT, **2016**.
